# Nitrate and Ammonium Affect the Overall Maize Response to Nitrogen Availability by Triggering Specific and Common Transcriptional Signatures in Roots

**DOI:** 10.3390/ijms21020686

**Published:** 2020-01-20

**Authors:** Laura Ravazzolo, Sara Trevisan, Cristian Forestan, Serena Varotto, Stefania Sut, Stefano Dall’Acqua, Mario Malagoli, Silvia Quaggiotti

**Affiliations:** 1 Department of Agronomy, Food, Natural resources, Animals and Environment, University of Padova, Agripolis—V.le dell’Università, 16, 35020 Legnaro (PD), Italy; laura.ravazzolo@unipd.it (L.R.); sara.trevisan@unipd.it (S.T.); cristian.forestan@unipd.it (C.F.); serena.varotto@unipd.it (S.V.); stefania.sut@phd.unipd.it (S.S.); mario.malagoli@unipd.it (M.M.); 2 Department of Pharmaceutical and Pharmacological Sciences, University of Padova—Via Marzolo 5, 35121 Padova, Italy; stefano.dallacqua@unipd.it

**Keywords:** ammonium, amino acids, gene expression, maize, nitrate, plant development, RNA-Seq, root

## Abstract

Nitrogen (N) is an essential macronutrient for crops. Plants have developed several responses to N fluctuations, thus optimizing the root architecture in response to N availability. Nitrate and ammonium are the main inorganic N forms taken up by plants, and act as both nutrients and signals, affecting gene expression and plant development. In this study, RNA-sequencing was applied to gain comprehensive information on the pathways underlying the response of maize root, pre-treated in an N-deprived solution, to the provision of nitrate or ammonium. The analysis of the transcriptome shows that nitrate and ammonium regulate overlapping and distinct pathways, thus leading to different responses. Ammonium activates the response to stress, while nitrate acts as a negative regulator of transmembrane transport. Both the N-source repress genes related to the cytoskeleton and reactive oxygen species detoxification. Moreover, the presence of ammonium induces the accumulation of anthocyanins, while also reducing biomass and chlorophyll and flavonoids accumulation. Furthermore, the later physiological effects of these nutrients were evaluated through the assessment of shoot and root growth, leaf pigment content and the amino acid concentrations in root and shoot, confirming the existence of common and distinct features in response to the two nitrogen forms.

## 1. Introduction

Nitrogen (N) represents about 2% of plant dry weight and strongly affects plant growth and crop productivity, being a structural constituent of proteins, nucleic acids and many secondary metabolites [1]. Since in natural environments, N availability is often limited, plants have developed specific mechanisms to adapt to different N sources [2]. Except for some species that can fix atmospheric nitrogen through symbiotic associations with soil microbes, most plants need to directly uptake nitrogen from the soil. Nitrate and ammonium are the two dominant inorganic N forms in natural and cropland soils, accounting for 70% of anion and cation plant absorption [3]. Nitrate represents the major N form in most aerated soils and ammonium is prevalent in acidic and/or water-saturated soils [1]. Due to the complex soil properties in soil waters, the diffusion coefficient of nitrate is 10–100-fold higher than that of ammonium [1]. Consequently, nitrate is rapidly transported towards the roots by mass flow, whereas ammonium is mostly adsorbed by soil particles [4]. To adapt to the fluctuations and different behavior in soil of both these ions and to optimize N uptake, plants need to adapt their root architecture by finely adjusting primary root (PR) and lateral root (LR) development. Several studies performed on *A. thaliana* [5,6,7,8,9] led to hypothesize that ammonium stimulates LR branching, whereas nitrate stimulates LR elongation, thus highlighting a complementary effect of these two ions on LR development which would reflect the plasticity of LRs to the distinct mobility of both N forms. However, although these two ions affect plant growth and physiology differently, concordant results were not always obtained, probably depending on the plant species and genotype utilized [10].

From the physiological point of view many similar responses, as for example trehalose synthesis, glycolysis and sucrose degradation, are triggered by both of them [11,12], but also different and specific signaling seem to exist in response to these nutrients [13]. Indeed, due to the higher need of energy required to assimilate nitrate, these two ions trigger contrasting effects on cellular energetics and redox status [14,15]. Furthermore, the presence of either nitrate or ammonium clearly affect the content of various metabolites and the activity of many enzymes [16,17,18,19,20,21]. Actually, metabolic and signaling responses to N provision also include redox homeostasis adjustments, which in turn may influence the global response to abiotic stress [22,23,24,25]. Over the past few decades, besides improving crop productivity [26], the intensification of the application of N fertilizers also triggered adverse and sometimes conflicting effects on plant–pathogen interactions [27] and plant disease development [28]. For these reasons, plant breeding for resistance also needs to carefully take into consideration plant-pathogen interactions under different N regimes [29].

Despite the different effect of nitrate and ammonium on plant metabolism and development are widely recognized, only a few studies have focused on the molecular characterization of plant responses to ammonium compared with those dealing with the nitrate regulation of gene expression [13].

The assimilation of ammonium and nitrate converges once that nitrate has been reduced to ammonium, leading us to suppose that genes commonly regulated by both ammonium and nitrate presumably respond to ammonium or to a metabolite downstream of ammonium assimilation (e.g., glutamate or glutamine) and that the nitrate-specific gene regulation depends on the nitrate ion itself or on nitrite [30] or on nitric oxide [31].

Nitric oxide has been identified as a crucial component of the signaling occurring in the transition zone of maize root after nitrate provision, being involved in the early developmental response of primary root to nitrate [32,33,34,35,36]. Moreover, more recent results on maize root [37] led us to hypothesize that the regulation of LR development by nitrate and ammonium could partially depend on the inhibition of strigolactones (SLs) biosynthesis observed in response to both these ions, but additional regulatory elements should be identified to better characterize common and specific mechanisms of the regulation of root development in responses to these two nutrients.

Untargeted approaches could strongly improve our knowledge on the pathways governing the physiological response to these two nutrients, allowing us to also identify novel modules of these responses. The identification of key genes underlying the response to nitrogen starvation and nitrate or ammonium provision may enable novel approaches to increase N use efficiency (NUE) and to improve plant resilience to nutritional stresses. In this study, an RNA-sequencing approach was applied to gain a comprehensive picture of common and specific transcriptional mechanisms underlying the early response to N-deprivation and nitrate or ammonium provision of young maize roots. Gene Ontology (GO) and MapMan enrichment analyses were also performed to identify the most important pathways that distinguish these responses. In addition, to better characterize the overall effects of these nutritional treatments on plant physiological status, biomass accumulation, chlorophyll, flavonoids and anthocyanins indexes and amino acid profiles of roots and leaves were also assessed later in the experiment. Our results provide new insight on the maize root molecular regulation of the early sensing of these nutritional cues, thus allowing to recognize key specific components which could impact on both root and whole plant development and physiology.

## 2. Results

### 2.1. Reads Processing and Differential Expression Analysis

RNA-Seq technique was employed to analyze the transcriptomic profiles of maize root apexes in response to N-supply as nitrate 1 mM (+NO_3_^−^) or ammonium (+NH_4_^+^), compared to those grown in N-deprivation (-N). After Illumina sequencing, adapters and low-quality reads were removed, resulting in 23 to 35 million high-quality reads per biological replicate, with about 98% of them mapped on the maize B73 reference genome (Table S1). Cuffdiff v2.2.1 [38] was then used for differential expression analysis after estimation of gene transcript abundances in the different conditions (expressed as RPKM—Reads Per Kb per Million) (Supplementary dataset 1).

**Figure 1:**
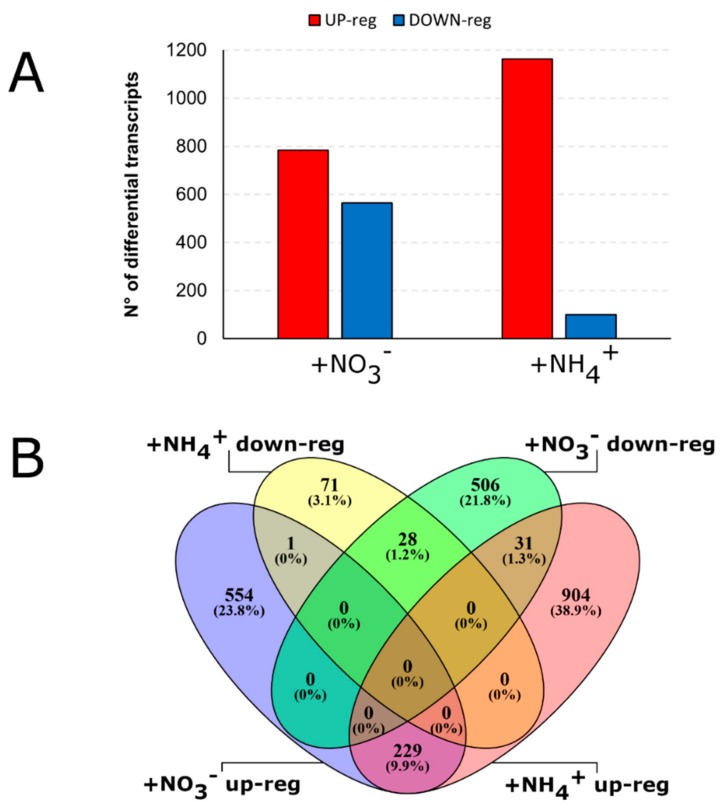
Distribution of differentially expressed genes (DEGs) identified (log2 FC >|0.58|; FDR ≤ 0.05) by RNA-Seq analysis from the comparison between NO_3_^−^ or NH_4_^+^ supplied maize seedlings for 24 h with respect to the control (-N, nitrogen deficient solution). Data are shown as number of genes differentially expressed in response to each treatments (**A**) and as Venn diagram (**B**). In (**A**), DEGs were classified as up-regulated according to their log2 fold change values (a log2FC threshold > |0.58| was set, corresponding to a 1.5 fold change increase or decrease in expression). In (B), the Venn diagram shows the numerical and percentage comparison of all significant up- and down-regulated differential expressed genes following +NO_3_^−^ and +NH_4_^+^ treatments. The no overlapping numbers represent the genes that are uniquely identified as DE in the corresponding treatment.

For each comparison between N-supply plants (+NO_3_^−^ or +NH_4_^+^) and N-deprived plants (-N), the genes showing a log2 fold change ratio >|0.58| (corresponding to a 1.5-fold change variation in expression level) and a false discovery rate (FDR)-adjusted *p*-value ≤ 0.05 were considered as differentially expressed genes (DEGs). Pairwise comparisons resulted in 1349 DEGs significantly responsive to NO_3_^−^, while 1264 were significantly regulated by NH_4_^+^ (Figure 1). The two applied N sources showed a different impact on the transcriptional regulation of root apexes: among the 1349 DEGs responsive to NO_3_^−^, 41.9% were up-regulated and 58.1% down-regulated, while among the 1264 DEGs responsive to NH_4_^+^, 92% were up-regulated and only 8% resulted in being significantly down-regulated by the cation (Figure 1A). The genes whose transcription was up- and down-regulated by each treatment were then grouped based on the magnitude of their transcriptional changes (Figure S1).

By comparing the DEGs regulated by nitrate or ammonium (Figure 1B), four sub-groups of genes were identified which expression was affected by both N sources. These groups only account for a small fraction of DEGS (289 genes, corresponding to the 12.4% of total DEGs). Among them, 229 DEGs were up-regulated in response to both NO_3_^−^ and NH_4_^+^, while 28 DEGs were commonly down-regulated in response to both NO_3_^−^ and NH_4_^+^. DEGs showing opposite transcriptional changes in response to NO_3_^−^ and NH_4_^+^ were identified as 32 DEGs. However, the majority of DEGs (87.6% of total DEGs) were specifically and exclusively regulated by nitrate (45.6% among the total DEGs) or ammonium (42% among the total DEGs) if compared with -N-treated roots, as shown in the Venn diagram.

**Figure 2:**
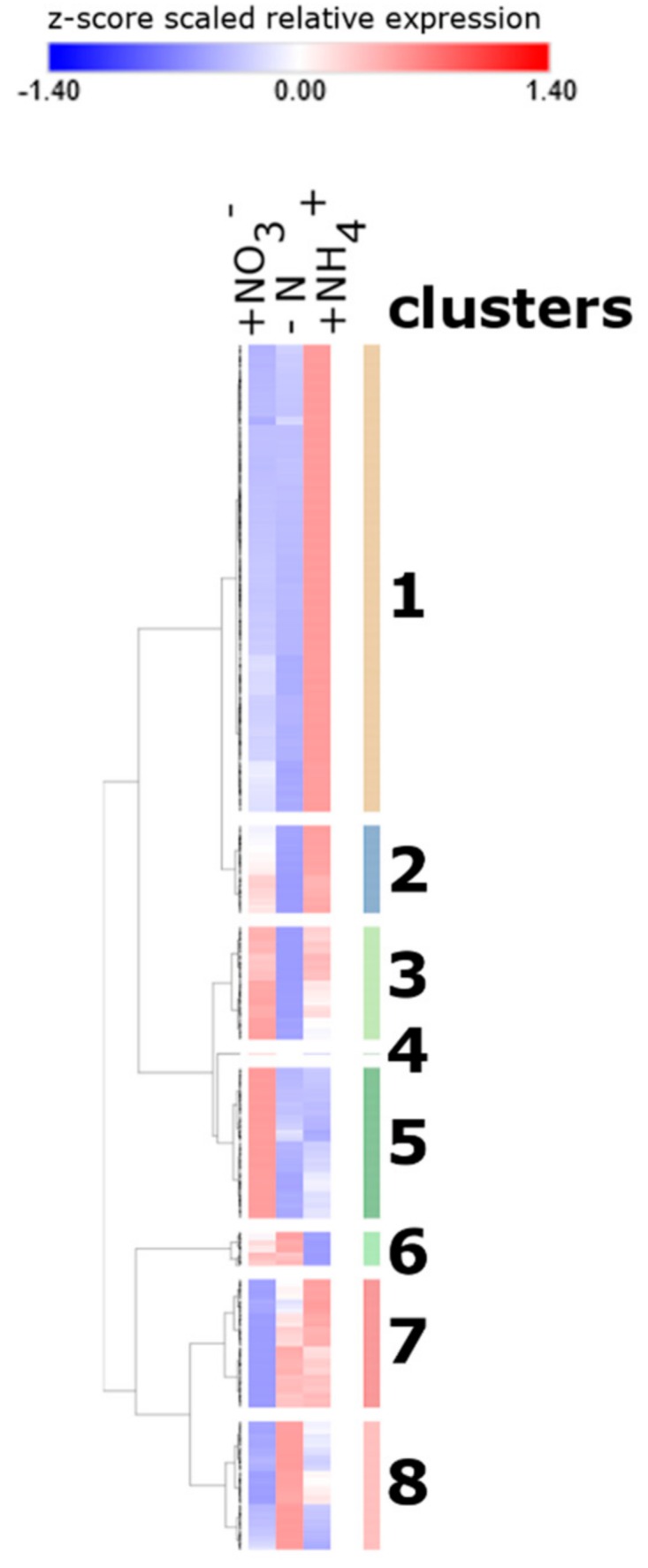
Clustering analysis of genes differentially expressed in +NO3- and +NH4+ treatments with respect to the control (-N) in maize root tissues. z-score scaled RPKM values for all the 2324 genes resulted as DEGs in at least one treatment were used for hierarchical clustering analysis. The analysis reveals eight different clusters with specific expression behaviors in response to different N-provision or deficiency. Re-scaled expression values for each gene in each sample are reported in a blue to red color scale (blue: lower RPKM values, red: higher RPKM values). RPKM: Reads Per Kb per Million.

To better dissect the overlapping and unique root response to 24 h of nitrate or ammonium provision, a hierarchical clustering of DEGs was then performed by using their normalized expression values in the three samples. The DEGs being differentially expressed in at least one treatment were arranged into eight clusters according to their expression dynamics, as displayed in the heat map (Figure 2; Table S2). As from the Venn analysis, the cluster analysis confirmed the existence of groups of genes commonly regulated by nitrate and ammonium provision (clusters 2, 3 and 8), while other are specifically regulated only by nitrate (clusters 5 and 7) or by ammonium (clusters 1 and 6). Cluster 4 contains a single gene up-regulated by nitrate and down-regulated by ammonium provision (Zm00001d015905). In addition, the heat map pointed out a different behavior within the genes up-regulated by both nitrate and ammonium, revealing 183 DEGs that are weakly up-regulated by nitrate but strongly up-regulated by ammonium (cluster 2) and 237 DEGs with a strong up-regulation induced by nitrate and a weak up-regulation induced by ammonium provision (cluster 3). Only a subset of the DEGs included in these two clusters were classified as commonly up-regulated by N supply in the Venn analysis.

### 2.2. Annotation and Classification of Clustered DEGs into GO Functional Categories

**Figure 3:**
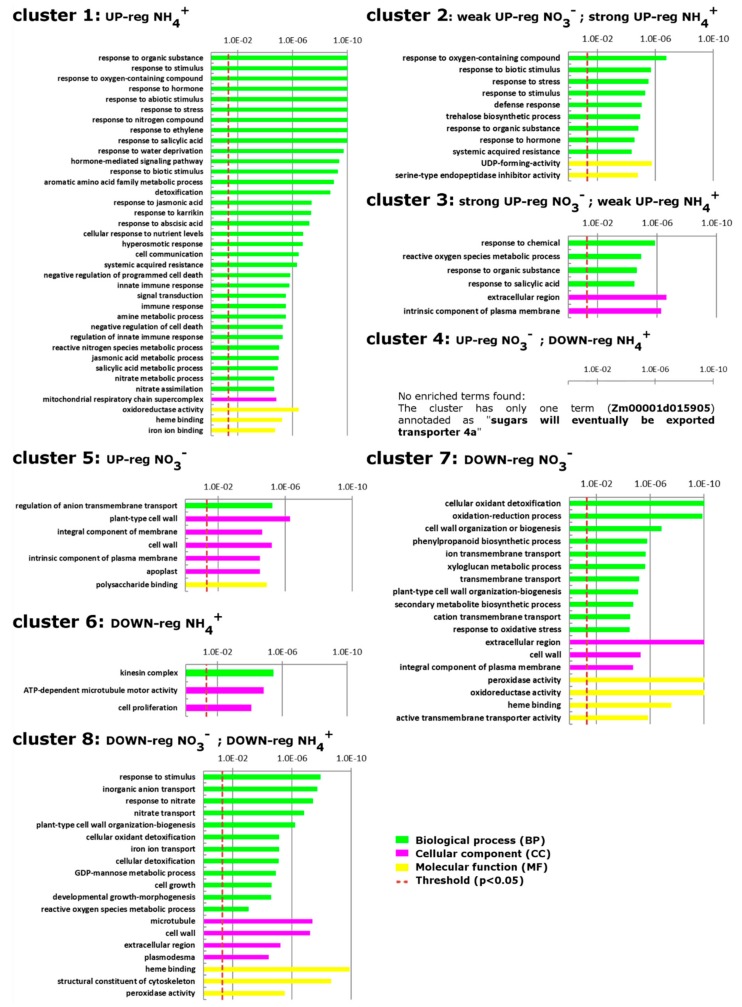
Enrichment analysis of DEGs clustered in 8 groups using Ontologizer. The figure shows the GO categories overrepresented among up and down-regulated gene sets in +NO_3_^−^ and +NH_4_+ treatments with respect to the control (-N) in maize root tissues.

A GO enrichment analysis was performed to functionally characterize the DEGs included in the eight different transcriptional profiles (Figure 3; Supplementary dataset 2; Table S3). Genes specifically up-regulated by ammonium (cluster 1) were enriched in GO terms related to ‘hormone-mediated signaling pathway’, in particular to ethylene, salicylic acid, jasmonic acid, karrikins and abscisic acid (Table S4). This term was also over-represented among DEGs included in cluster 2, which included those genes strongly up-regulated by NH_4_^+^ and weakly up-regulated by nitrate (Table S6). It is interesting to note how these hormones could be linked for their functions to the other main terms induced by ammonium, such as ‘response to biotic stimulus’ (Table S4). Indeed, ammonium up-regulated DEGs were also enriched in terms involved in the ‘immune response’ and “response to water deprivation”. Conversely, among the very few terms enriched in DEGs of cluster 6 (down-regulated by ammonium), ‘cell proliferation’ was unique.

Among the GO terms associated with NO_3_^−^ response (Table S5), the terms “polysaccharide binding” and “apoplast” were over-represented among DEGs included in cluster 5 (up-regulated only by nitrate). Regarding the terms enriched in DEGs repressed by NO_3_^−^ (cluster 7), many terms could be related to the transmembrane transport, such as “cation transmembrane transport”. In addition, other enriched terms specific of this cluster are related to secondary metabolism, such as “secondary metabolite biosynthetic process”.

Investigating the common response to both nitrate and ammonium (Table S6), terms regarding cytoskeleton organization, such as “structural constituent of cytoskeleton”, and ROS metabolism, such as “reactive oxygen species metabolic process”, are enriched in cluster 8 (DEGs down-regulated both by NH_4_^+^ and NO_3_^−^). Finally, the single gene induced by nitrate and repressed by ammonium (cluster 4) corresponds to the Zm00001d015905 accession, which encodes a SWEET sugar transporter.

These results indicate that NH_4_^+^ strongly and positively affects the hormones involved in the stress response. In addition, ammonium down-regulated genes related to “cell proliferation”. On the other hand, nitrate repressed the expression of genes related to the cation transmembrane transport and secondary metabolism, while only a few terms appeared specific for the nitrate-induced up-regulation, above all the apoplast localization and the polysaccharide binding. Moreover, genes down regulated by both inorganic N sources are associated to reactive oxygen species (ROS) detoxification and cytoskeleton reorganization.

### 2.3. Classification of DEGs into MapMan Functional Categories

**Figure 4:**
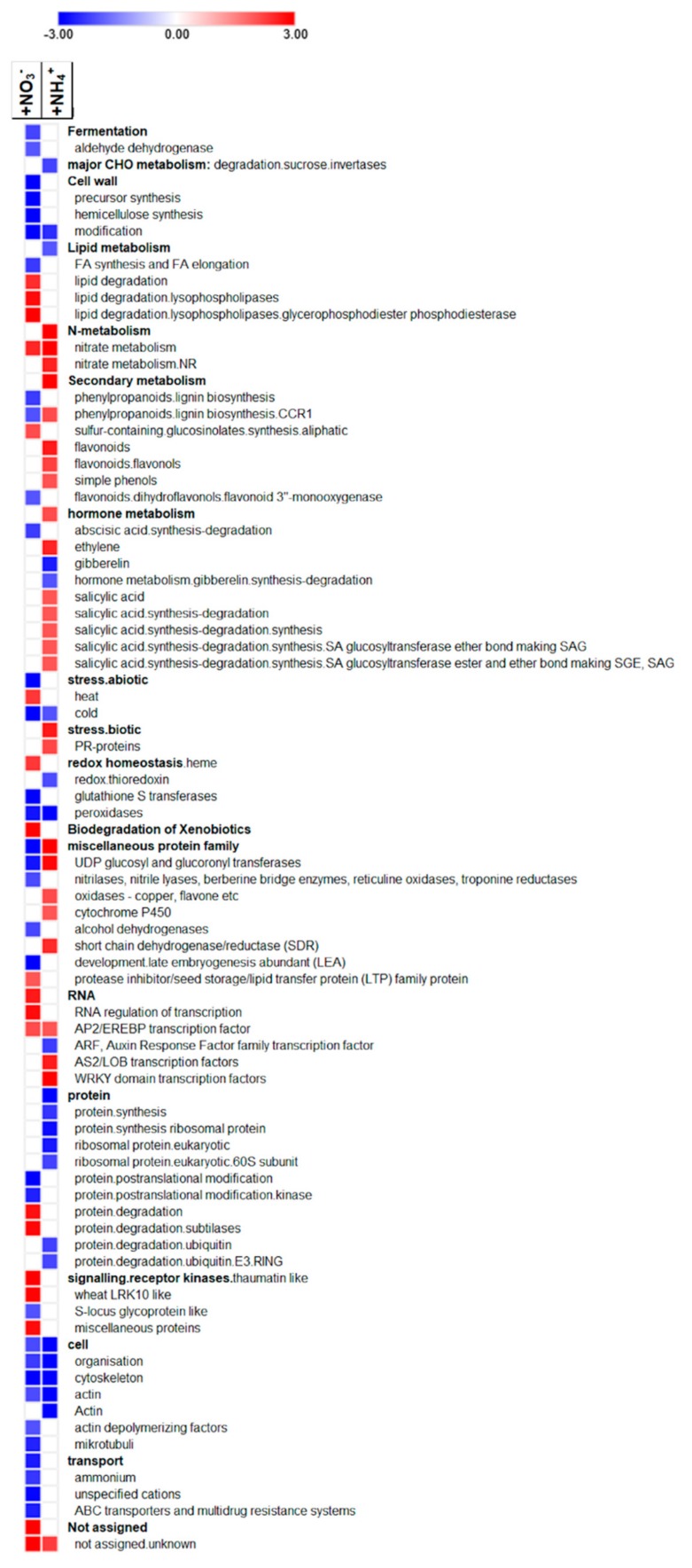
MapMan functional categories enriched of DEGs identified in two treatments (+NO_3_^−^/-N; +NH_4_^+^/-N). Z-scores were deduced from p-values (i.e., 1.96 corresponds to a P-value of 0.05) and are mapped in a blue to red color scale (blue: enrichment among down-regulated DEGs, red: over-representation among up-regulated DEGs).

In addition to GO enrichment, functional analyses of DEGs were performed by means of MapMan over-representation analysis. Several pathways were found enriched or depleted among genes differentially expressed in the two analyzed N-treatments (Figure 4). However, as expected, some gene categories were similarly enriched following the application of the two N-treatments. The MapMan categories “N-metabolism” and “cell organization” were highly affected by both NO_3_^−^ and NH_4_^+^. The first bin was significantly enriched among genes up-regulated by both NO_3_^−^ and NH_4_^+^, while the second bin—in particular annotations related to the cytoskeleton organization through actin and tubulin—was strongly over-represented among genes down-regulated in response to both these N forms. Moreover, the bin ‘cell wall’ was significantly over-represented among genes down-regulated by NO_3_^−^ provision, while this pathway was not significantly affected by NH_4_^+^.

Among the ‘regulation of transcription’ bin, the AS2/LOB and WRKY transcription factor (TF) families appeared enriched exclusively in ammonium up-regulated genes, while AP/EREBP TF family was also over-represented among nitrate-induced genes.

In addition, genes associated to the ‘hormone metabolism and responses’ bin were differentially regulated by both nitrate and ammonium, with this last being more effective, as already shown from GO enrichment. “Ethylene” and “salicylic acid metabolism”’ bins were over-represented among DEGs induced by NH_4_^+^, while “gibberellin metabolism” and “abscisic acid degradation” bins—over-represented among genes—were repressed by the ammonium and nitrate, respectively.

Moreover, NO_3_^−^ was shown to specifically induce over-representation of the “lipid degradation” bin, thus adding new data to the few obtained with GO enrichment. Among the “secondary metabolism” bin, genes associated with “sulphur metabolism” sub-category were over-represented in NO_3_^−^ up-regulated DEGs, ‘flavonoids’ sub-category was enriched among NH_4_^+^ up-regulated ones, while “lignin metabolism” was significantly enriched between genes down-regulated by nitrate. In addition, NH_4_^+^ induced strong enrichment of DEGs in the ‘biotic stress’ bin, thus confirming GO enrichment output.

### 2.4. Differences in Biomass Accumulation between Ammonium and Nitrate Nutrition

**Figure 5:**
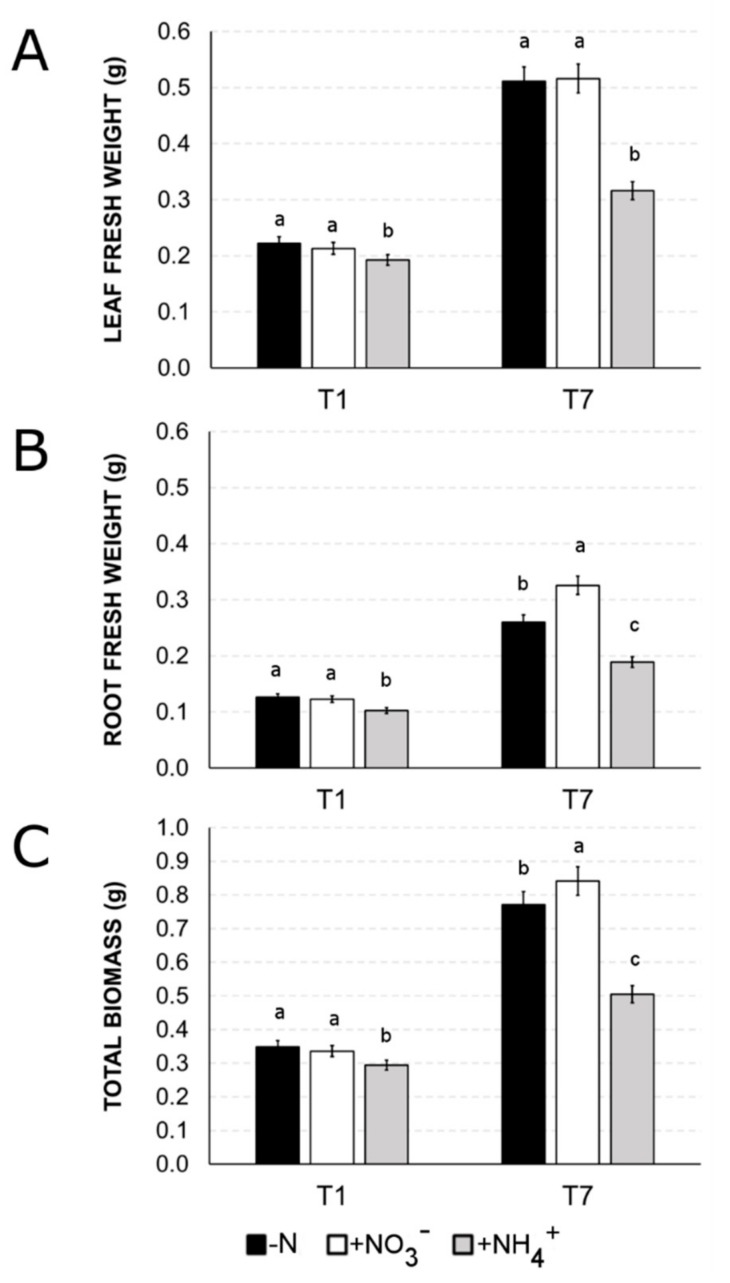
Effect of different N-source (NO_3_^−^ or NH_4_^+^) or N-deficiency (-N) on leaf biomass (fresh weight, panel **A**) and root biomass (fresh weight, panel **B**). The distribution of total biomass was reported in panel **C**. T1 shows the effect after 24 h of treatment, T7 after 7 days. Similar letters at the top of the bars are not significantly different (*p* < 0.05) by an ANOVA test. Each value is designed as the mean of three biological replicates ± SE.

The estimation of plant growth, measured as the biomass of roots and leaves during the different nutritional treatments, revealed that the two nitrogenous sources affect plant growth with a different dynamic (Figure 5). After 24 h of treatments (T1), no significant differences were detected among both shoot and root biomass allocation in nitrogen-starved plants (-N, negative control) and nitrate-supplied plants (+NO_3_^−^), while ammonium-(+NH_4_^+^) early triggered a significant reduction in total biomass accumulation with respect to both -N and +NO_3_^−^ plants (about −16%) (Figure 5C).

After one week of nitrogen provision (T7), the total biomass accumulation was statistically differentiated in the three sets of plants (Figure 5C). The highest accumulation was observed in +NO_3_^−^ plants that reached a total biomass 10% higher in comparison to that measured for -N plants. This increase was mainly due to a higher root biomass accumulation (+25% of the root biomass compared to -N-plants), while shoot biomass was similar to -N (Figure 5A,B). Ammonium application resulted in lower total biomass accumulation with respect to both nitrogen-starved plants (-N; −35%) and nitrate-supplied plants (+NO_3_^−^, −40%), and the reduction was detectable in both leaves (about −39% with respect to both -N and +NO_3_^−^,) and roots (−28% if compared to -N, and −42% if compared to +NO_3_^−^).

**Figure 6:**
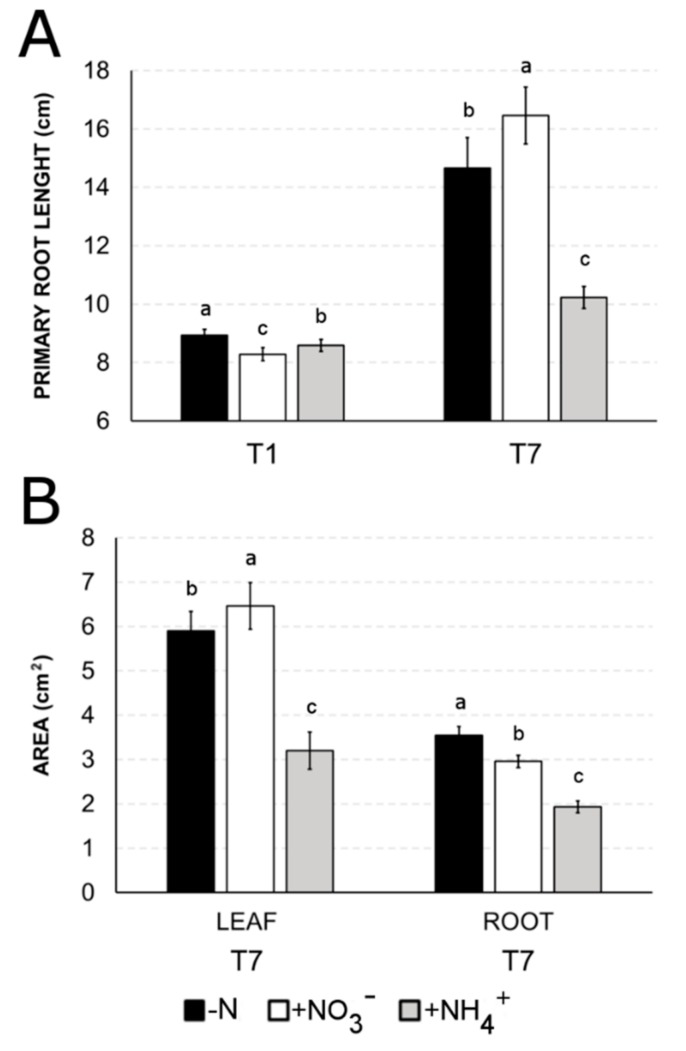
Effect of different N-source (NO_3_^−^ or NH_4_^+^) or N-deficiency (-N) primary root length (cm, panel **A**) and surface area (cm^2^, panel **B**). T1 shows the effect after 24 h of treatment, T7 after 7 days. Surface area was obtained for both roots and leaves only at T7. Similar letters at the top of the bars are not significantly different (*p* < 0.05) by an ANOVA test. Each value is designed as the mean of three biological replicates ± SE.

Primary root (PR) length showed a different trend between T1 and T7, with the longest PR in -N at T1, while at T7 a different behavior of the two N-sources was observed (Figure 6A). Indeed, after seven days the longest PR was induced by nitrate (+12% compared to -N), while ammonium provision induced a strong reduction in PR length (−30%) with respect to -N. Nitrate significantly induced a higher leaf area (+10%) but a smaller root area (−17%) if compared to N-deprived plant (Figure 6B). Considering leaves and roots area at T7, NH_4_^+^-treated plants showed a 46% reduction in both parameters with respect to -N plants.

### 2.5. Leaf Pigments Prediction with Optical Sensor

**Figure 7:**
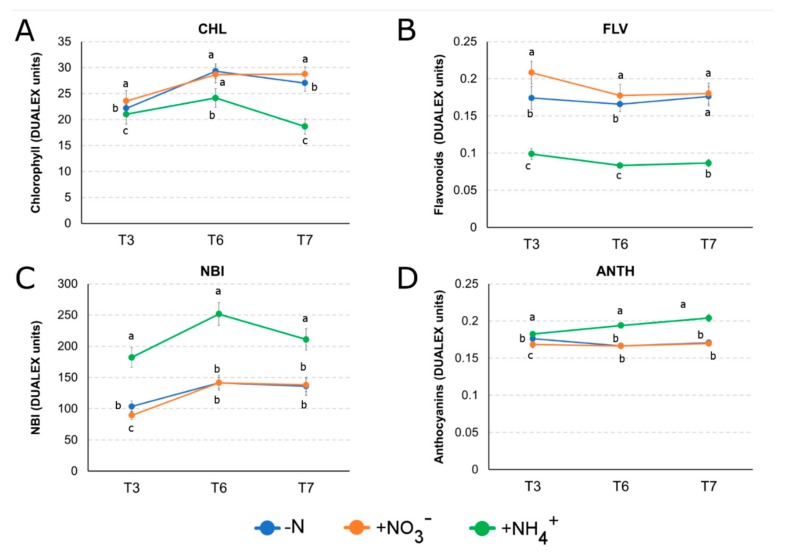
Values of (**A**) leaf chlorophyll content, (**B**) leaf flavonoids content, (**C**) leaf anthocyanins content and (**D**) Nitrogen Balance Index. Maize seedlings were grown 24 h in a N-deprived nutrient solution and then transferred to a 1mM N-supplied media (NO_3_^−^ or NH_4_^+^) or to a N-deprived solution (-N) for additionally 3 days (T3), 6 days (T6) and 7 days (T7). Epidermis absorbance was obtained with the optical sensor DUALEX SCIENTIFIC+^TM^ (Force-A) at the same hour of every time point. Values represents means of three replicates ± SE. Similar letters at corresponding dot within treatments are not significantly different (*p* < 0.05) by an ANOVA test.

To study the later effect in planta of the absence or supply of N as inorganic ions, the leaf pigment contents were indirectly measured through the optical sensor Dualex. Three fluorescence indices, namely leaf chlorophyll (CHL), flavonoids (FLAV) and anthocyanins (ANTH), together with the ratio between CHL and FLAV which is called NBI (Nitrogen Balance Index), were evaluated in the plants subjected to the three different N treatments at three times points: T3 (3 days), T6 (6 days) and T7 (7 days) (Figure 7). NO_3_^−^ induced the strongest accumulation of CHL after 3 days of treatment, whilst N-starved (-N) and NH_4_^+^ supplied plants showed slight but significant differences (Figure 7A). Both -N and +NO_3_^−^ plants showed a similar increasing trend of CHL accumulation, with a maximum at T6 where it appeared statistically identical, while 24 h later (T7) it was slightly but statistically higher in +NO_3_^−^ plants than in -N plants. On the other hand, ammonium provision (+NH_4_^+^) determined only a moderate CHL accumulation at T6, while a drastic reduction was visible at T7.

Regarding flavonoids index (FLAV), a decreasing trend of accumulation was observed for nitrate-supplied plants, with the highest content at T3 (Figure 7B). N-deficient plants showed a similar but significant lower level until T6, while at T7 FLAV index became identical between +NO_3_^−^ and -N. As for CHL, ammonium supplied plants showed a lower FLAV content compared to the others two sets of plants, but this pattern of accumulation remained stable at T6 and T7 too.

The anthocyanin accumulation (ANTH) (Figure 7D), showed a constant and increasing higher level in +NH_4_^+^ plants from T3 to T7, while +NO_3_^−^ and -N treatments seemed to not affect ANTH level at any time point.

NBI index was strongly enhanced by the provision of ammonium to the plants from T3 to T6, but the value dropped at T7 (Figure 7C). The same NBI positive trend was observed in both nitrate-supplied and nitrogen-starved plants from T3 to T6, but the value stayed stable and indistinguishable between the two treatments from T6 to T7. However, the NBI index exhibited by both the +NO_3_^−^ and -N plants was lower than the one observed in the +NH_4_^+^ plants at all the time points.

### 2.6. Nitrate and Ammonium Differently Regulate the Amino Acid Profile in Maize Root and Leaf

**Figure 8:**
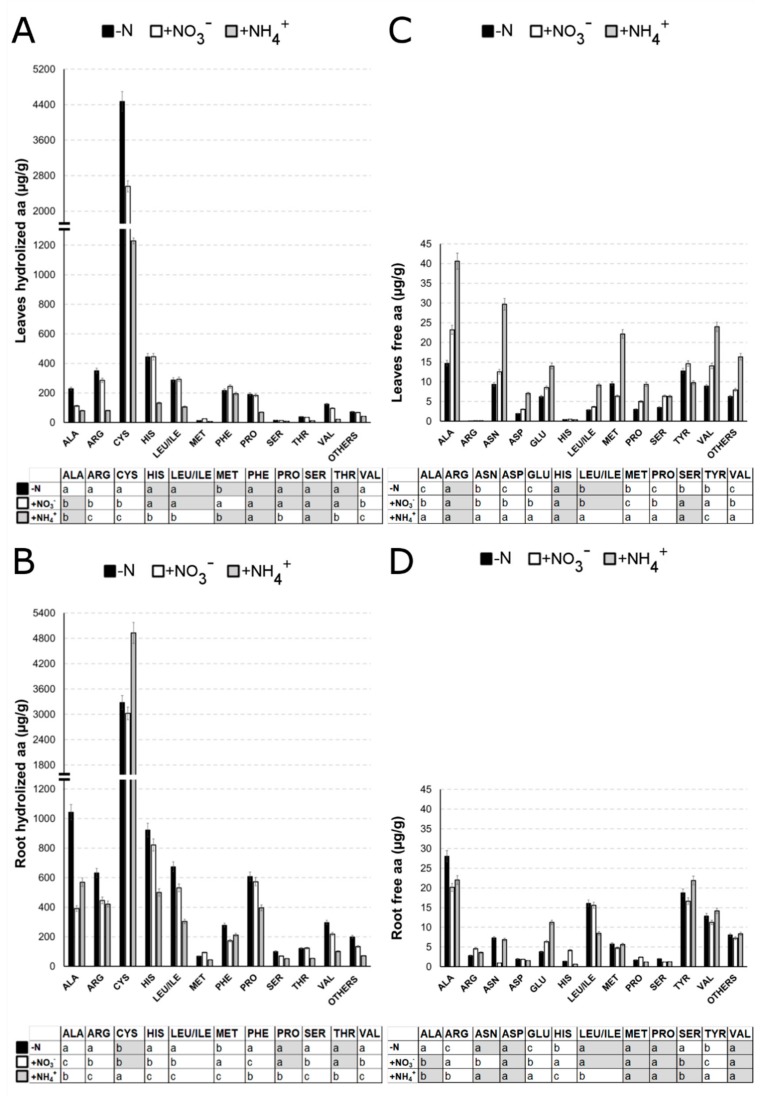
Levels of the mostly abundant hydrolysed amino acid (**A**,**B**) and free amino acid (**C**,**D**) with respect to leaves (**A**–**C**) and roots (**B**–**D**). Maize seedlings were grown 24 h in a N-deprived nutrient solution and then transferred to a 1mM N-supplied media (NO_3_^−^ or NH_4_^+^) or to a N-deprived solution (-N) for additionally 7 days. Amino acids levels were detected by means of HILIC-MS/MS. For each panel the results of ANOVA test (*p* < 0.05) are reported and similar letters within treatments show no significant differences. Each value is designed as the mean of three biological replicates ± SE.

In order to appreciate the later effects of the three nutritional treatments on plant metabolism, total free amino acids and total hydrolyzed amino acids were determined in root and leaf tissues after 7 days (Figure 8).

A significant lower global free amino acid level in the root was observed upon NO_3_^−^ (−12.6%) and NH_4_^+^ (−4.5%) treatments in comparison to the amount detected in N-deficiency. On the contrary, higher concentrations of global free amino acid were observed in leaves upon both NO_3_^−^- (+28.5%) and NH_4_ (+124.9%) compared to -N (Table S7). The total hydrolyzed amino acid content in the leaves and roots was, instead, always lower in nitrate or ammonium-supplied plants if compared with N-deprived plants, especially in leaves of NH_4_^+^-supplied seedlings which showed a decrease in hydrolyzed amino acids of about 70% compared to -N plants (Table S8).

Among the free amino acids, alanine (Ala) was the most abundant one, representing about 17–25% of the total amount of free amino acids found in leaves and roots for all treatments (Table S7). It showed a higher accumulation upon both nitrate or ammonium supply in both tissues. This effect was particularly evident in the case of ammonium provision (Figure 8C,D).

Contrariwise, the most abundant among the hydrolyzed amino acids was cysteine (Cys) which reached, or even exceeded, the 50% of the total hydrolyzed amino acids amount in all tissues and all treatments ((Figure 8A,B, Table S8), followed by histidine (His) and arginine (Arg). Cys showed the greatest accumulation at root level treated with NH_4_^+^, while the NO_3_^−^ treatment induced no significant differences in Cys concentration compared with -N. Oppositely, Cys level was highest in leaves subjected to N-deficiency, while it was 40% lower in NO_3_^−^ -supplied plants and even 70% lower in +NH_4_^+^- supplied plants) (Figure 8A,B).

Regarding the glutamine (Gln)-free level, no significant differences among treatments were detected in the leaves nor the roots, while a significant higher level in free glutamic acid (Glu) was observed both in roots and leaves treated with NO_3_^−^ and NH_4_^+^ in comparison to -N-roots. Notably, in roots, the free Glu level almost doubled with +NH_4_^+^ if compared to NO_3_^−^ treatment, while only a very low amount of this amino acid was detected after hydrolysis.

Furthermore, free asparagine (Asn) displayed higher accumulation in response to NH_4_^+^ in leaf, while no significant differences were detected upon NO_3_^−^ supply. On the contrary, in roots, Asn displayed no significant differences between -N and NH_4_^+^, while a huge drop in response to NO_3_^−^ was observed.

Free arginine (Arg) and histidine (His) were more abundantly represented in NO_3_^−^-supplied roots, while they were not detectable in any treatment at leaf level. After hydrolysis, Arg showed the highest level both in roots and leaves of -N seedlings in comparison to N-supplied plants. However, NH_4_^+^-provided seedlings evidenced the lowest amount of Arg. On the other hand, hydrolyzed His showed no significant differences between -N and + NO_3_^−^-treatments in leaves, while NH_4_^+^ treatment reduced its content by half. In root, hydrolyzed His displayed the same trend as that described above for hydrolyzed Arg.

Furthermore, the content of free aspartic acid (Asp), methionine (Met), proline (Pro) and valine (Val) was significantly higher in leaves of NH_4_^+^-treated plants whereas no changes were evidenced in roots in response to any treatment. After hydrolysis, Met was the only amino acid that showed the highest amount in response to NO_3_^−^ (and not to -N) in both leaves and roots, while Pro and Val displayed the highest amount in response to N-deficiency in roots.

Finally, free tyrosine (Tyr) content was lower in NH_4_^+^-treated leaves and slightly higher in the leaves of NO_3_^−^ supplied seedlings, while in the roots this trend appeared opposite. After hydrolysis, Tyr level was not detected, while phenylalanine (Phe) showed a significant higher level in response to N-deprivation in root.

In conclusion, NH_4_^+^ supplied plants generally displayed higher levels of t free amino acids in leaves (above all for Ala, Asn, Glu, Met and Val), with the only exception of Tyr being more abundantly represented in leaves of NO_3_^−^ treated plants. In roots, nitrate and ammonium provision similarly affected the accumulation of free Ala, Asp, Met, Pro and Val, while Arg and His were higher in NO_3_^−^-treated roots. On the other hand, Ala and Ser were the only free amino acids showing the highest root content in response to N-deprivation (-N). As far as hydrolyzed amino acids are concerned, they displayed the lowest content in NH_4_^+^-supplied plants and were instead most abundant in the tissues of N-deficient plants (-N). Among them, Met was the only one that accumulated more in response to NO_3_^−^ both in leaves and roots.

## 3. Discussion

Plant development is highly influenced by nitrogen (N) availability, so specific transport and signaling mechanisms were evolved in response to different N sources [2]. Most of the studies have been focused on nitrate (NO_3_^−^) and ammonium (NH_4_^+^), since they are present in both natural and cropland soils at much higher levels than other sources [1]. Plants supplied with ammonium or nitrate display many physiological differences, for example contrasting effects on cellular energetics and redox status [14,15]. A better knowledge of mechanisms regulating nitrate and ammonium response in plants will help to develop a strategy to enhance inorganic N use efficiency (NUE), leading to decrease the use of N fertilizers [39].

Previous results [37] led to hypothesize that nitrate and ammonium both inhibit strigolactone (SL) exudation by maize seedlings, even though to a different extent. In the same paper it was also postulated that this inhibition of SL production could at least partially regulate the development of lateral root (LR). However, it is known that nitrate and ammonium trigger LR growth by activating different mechanisms which are likely dependent by specific molecular events. To better characterize this response an untargeted approach was applied to deepen the early transcriptomic signature of maize roots in response to N-depletion, nitrate provision or ammonium provision. Results obtained confirmed that both nitrate and ammonium significantly down-regulate the expression of genes involved in the strigolactone biosynthesis (Supplementary dataset 1). In addition, a significant up-regulation of the transcription of some well-known genes involved in N transport and metabolism in response to both these ions was noticed (Supplementary dataset 2, Table S3, Figure 4). These preliminary evidences support the correctness of the utilized rationale.

Ammonium provision to N-starved roots predominantly up-regulates gene transcription, with only very few genes being down-regulated, whilst nitrate supply induces and represses transcription to a similar extent (Figure 1). Other studies performed in rice [40,41] and in oilseed rape (*Brassica napus*) [10], even if with longer treatments, showed a prevalent down-regulation of gene expression in response to ammonium, suggesting the existence of individual responses among genotypes and in dependence of the duration of the treatment.

Our results also point out that a huge percentage of genes are specifically responsive to only one of these two nitrogen forms, suggesting that distinct and independent pathways are activated to optimize the plasticity of the response to these two ions, as already showed in various species [9,42,43,44,45]. In particular, Gene Ontology (GO) (Figure 3, Table S4–S6, Supplementary dataset 2) and MapMan (Figure 4) enrichments clearly indicate that nitrate provision to N-deprived roots negatively affect transmembrane transport and secondary metabolism, suggesting that 24 h of nitrate provision are sufficient to down-regulate many root saturable transport systems [46,47] and to slow down the biosynthesis of secondary metabolites such as phenylpropanoids and flavonoids, which are known to be abundantly synthetized in the stress response signaling [48,49,50]. Nitrate also specifically induces the transcription of genes involved in polysaccharide binding and sugar transport, congruent with results on leaf area, biomass accumulation and chlorophyll index (Figure 5, Figure 6 and Figure 7) and with previous studies [51,52,53].

Ammonium provision seems to specifically activate genes involved in the hormonal signaling underlying defense responses, leading to suppose that it might positively affect the tolerance to biotic stress (Figure 3 and Figure 4), confirming that the N form provided could potentially affect the degree of plant resistance [54,55,56].

Current results also highlight specific and exclusive regulation of unique family of transcription factors in response to ammonium (Figure 4), which noticeably induces the expression of genes encoding WRKY transcription factors, playing crucial roles in the response to stress [57,58,59]. Moreover, ammonium triggers the activation of genes belonging to pathways related to water deprivation (Figure 3), which together with chlorosis, growth inhibition and decreased root: shoot ratio, has been hypothesised to represent a distinguish feature of the response to NH_4_^+^ depending on its toxicity [60].

On the other hand, nitrate specifically down-regulates the transcription of genes related to hemicellulose synthesis and transport (Figure 4). Accordingly, similar expression profiles have been recently evidenced in Arabidopsis [61] and rice [62], suggesting that cell wall remodeling might be important for the enhanced nitrate uptake and a correct plant growth. Nevertheless, the exact molecular mechanism by which the N status affects cell wall modelling is still unknown [63].

Besides those characterizing specific responses to nitrate or ammonium, further groups of genes displayed a common trend of regulation in response to both ions. Genes encoding key elements in the pathway leading to reactive oxygen species (ROS) detoxification were mainly down-regulated by both these N sources, as a consequence of the recovery of a balanced nutritional equilibrium. Furthermore, both nitrate and ammonium induce the expression of genes encoding transcription factors belonging to the AP2/EREBP family (APETALA2/Ethylene-responsive element binding protein). The identification of common elements belonging to shared signaling pathway similarly regulated upon nitrate and ammonium, confirms the existence of many processes responsive to the overall plant nitrogen status as also observed by Wang et al. [11] and Scheible et al. [12]. Genes regulated in a coordinated way by both ammonium and nitrate presumably respond to a signal downstream of ammonium assimilation, as for example glutamate (Glu) and glutamine (Gln), which have been demonstrated to be powerful regulators of gene expression [64].

Biomass accumulation, primary root growth and chlorophyll accumulation are strongly compromised in response to the prolonged supply of ammonium (Figure 5A, Figure 6A and Figure 7A). On the contrary, nitrate positively influences the root biomass production (Figure 5), primarily thanks to the induction of longer and thinner roots [65].

After few days of NH_4_^+^ supply, a higher content of anthocyanins was also observed (Figure 7D). Anthocyanins are secondary metabolites derived from the specific branch of the flavonoid pathway and key molecules in the defense against environmental stresses [66]. A greater accumulation of free amino acid in the leaves of NH_4_^+^-provided plants was also noticed (Figure 8C,D). The increase in amino acids content in leaves in response to ammonium has been already documented and interpreted as a toxic symptom [60], but it has also been correlated with an enhanced abiotic stress tolerance through the induction of protecting pathways [67]. For instance, ammonium induced a higher amount of proline both in *Matricaria chamomilla* [68] and *Oryza sativa* leaves [69]. In the present study, the amount of free proline doubled in response to ammonium treatment in leaves, when compared to nitrate-supplied plants (Figure 8C). Proline accumulation is known to act as an osmolyte and chaperone that is accumulated under various stress conditions [70], with a positive correlation with an enhanced stress tolerance to high ammonium conditions in rice [69].

Compared with nitrate-provided plants, ammonium-supplied plants also displayed a higher level of free asparagine in both leaves and roots, possibly due to the asparagine involvement in the mechanism of cell protection against NH_4_^+^-induced stress [71,72]. Furthermore, asparagine has been suggested to play a role in the response to many abiotic stresses, for example sulphur- and phosphate-deficiency, together with drought and salt stress [73,74]. Moreover, N can be redirected from glutamine to asparagine as a temporary measure to control excessive ammonium provision [75]. These results altogether further support the idea that ammonium nutrition could enhance stress tolerance, perhaps as a secondary effect of its slight toxicity, even though the mechanism(s) underlying this toxicity are not still completely understood [76,77].

Furthermore, levels of free glutamate detected would confirm the recognized role of this amino acid [78,79] as a putative regulatory signal accumulating in both nitrate and ammonium-provided roots (Figure 8). However, glutamine was detected at very low levels in maize roots and displayed higher concentration in N-starved roots. Regarding the hydrolyzed amino acid profile (Figure 8A,B), ammonium induced a global lower hydrolyzed amino acid level in roots if compared to N-deprived plants (Figure 8A,B), with only cysteine being increased of a 50%. Cysteine can be used as an extra energy source for plant [80] but also to produce sulphur-containing defense metabolites such as glutathione [81]. These findings are coherent with the activation of pathways related to oxidative and biotic stress response upon ammonium provision, as shown in the enriched GO categories of cluster 1 (Figure 3, Supplementary dataset 2).

Nitrate-supplied plants showed a significant increase in the content of hydrolyzed histidine (His) in roots (Figure 8D). Thanks to the presence of an imidazole side group, histidine can be found in the active site of many enzymes. Moreover, histidine plays important roles in phosphoryl transfer and metal ion homeostasis [82,83]. Accordingly, many genes down-regulated by nitrate showed enriched GO terms related to metal ion transport and response (Figure 3, Supplementary dataset 2). Nevertheless, the routes of histidine biosynthesis and catabolism in plants are still scarcely understood, so future research should be aimed at deepening the link between nitrate-supply and increased histidine content in root.

Nitrate also led to a higher accumulation of hydrolyzed methionine both in roots and leaves (Figure 8A,B). Methionine is a precursor of the primary methyl group donor S-adenosyl-methionine (SAM). SAM is the precursor of ethylene, and it is also involved in the regulation of cell division and the synthesis of cell wall, cell membrane and chlorophyll [84]. This result is in accordance with the chlorophyll content in the leaves of nitrate-provided plants (Figure 7) and with the effects of nitrate on the transcription of genes involved in cell wall biosynthesis, as revealed by MapMan and enrichment analysis in roots (Figure 3 and Figure 5, Supplementary dataset 2), leading us to suppose that nitrate-supplied plants need to balance between cell wall loosening and thickening [85], leaf pigments content [52] and secondary metabolites production [50] to allow for correct plant growth in response to enhanced N uptake.

## 4. Materials and Methods

### 4.1. Maize Seedlings Growth

Seeds of maize (*Zea mays* L.) B73 inbred line were rinsed in distilled water and germinated on wet filter paper at 25 °C in the dark, as described by Manoli et al. [35]. After 3 days, germinated seedlings were pre-treated for 24 h in a N-deprived solution and then transferred to: -N (negative control), NO_3_^−^ 1 mM or NH_4_^+^ 1 mM supplied complete nutrient solution [37]. RNA extraction for RNA-Seq analysis was performed after 24 h (T1) in each treatment solution. The fresh weight and primary root (PR) length of the leaves and roots were obtained after 24 h (T1) and 7 days (T7). At T7, the amino acid content of root and leaves was determined, and the area of leaves and roots was also measured. Chlorophyll, flavonoids and anthocyanins index were measured after 3 days (T3), 6 days (T6) and 7 days (T7) in each condition. The nutrient solutions were constantly aerated and changed every two days. Seedlings were grown in a growth chamber with a day/night cycle of 14/10 h at 25/18 °C air temperature, 70%/90% relative humidity, and 280 μmol m^−2^·s^−1^ photon flux density.

All sampling or measurements were performed at 11 a.m. after 4 h of light.

For each condition, three biological replicates were analyzed (20 plants for each condition).

### 4.2. RNA Extraction and Libraries Preparation for Illumina Sequencing

Lengths of 1.5 cm of root apices from the root tip cap were sampled from 20 pooled seedlings at T1 in three independent biological repetitions, and immediately frozen in liquid nitrogen. Total RNA was extracted using TRIzol reagent (Invitrogen, Thermo Fisher Scientific, Waltham, MA USA) and a subsequently DNAse digestion was performed whit RQ1 RNAse-free DNAse (Promega, Milano, Italy) on an aliquot of total RNA as described by Trevisan et al. [32]. The extracted RNA was quantified using a Nanodrop 1000 (Thermo Scientific, Nanodrop Products, Wilmington, DE, USA) and its quality further validated by a Fluorometer analysis (Thermo Scientific, Qubit^®^ 2.0, Wilmington, DE, USA). Libraries for Illumina sequencing were prepared according to the manufacturer instructions using the TruSeq RNA Sample Preparation kit (Illumina, San Diego CA, USA). Sequencing was performed at the Centro di Ricerca Interdipartimentale per le Biotecnologie Innovative (CRIBI, Padova, Italy), on a NextSeq500 (Illumina) instrument.

### 4.3. Processing of Sequencing Reads and Differential Expression Analysis

Base calling was performed using the Illumina Pipeline. The resulting raw reads (23–35 million per library; Table S1) were processed for adapter clipping and quality trimming using Trimmomatic 0.33 [86]. The resulting high-quality reads were mapped to the maize B73 reference genome (RefGen ZmB73 Assembly AGPv4 and Zea_mays.AGPv4.38.gtf Gramene transcript annotation) [87] with Tophat 2.0.13 [88] using the following modifications from default parameters: maximum intron size, 60,000; minimum intron size, 5; up to three mismatches and gaps allowed. The sequence alignment files (BAM format) were then filtered using Samtools [89] to remove alignments with MAPQ smaller than 1 (corresponding to multi-mapped reads assigned to more than 10 different genomic positions). Differential expression analyses were performed with Cuffdiff v2.2.1 [38] selecting the following options: -multi-read-correct, -compatible-hits-norm, -dispersion-method per-condition and -library-norm-method quartile. The genes showing a false discovery rate (FDR)—an adjusted *p* value ≤ 0.05—were considered as differentially expressed genes (DEGs). Hierarchical clustering of DEGs was performed by average linkage and Pearson distance using the Morpheus software (https://software.broadinstitute.org/morpheus/) [90] and displayed as a heat map. RNA-Seq data from this article can be found in the Gene Expression Omnibus data library under accession number GSE141860 (https://www.ncbi.nlm.nih.gov/geo/query/acc.cgi?acc=GSE141860) [91].

### 4.4. Gene Ontology (GO) Enrichment and Functional Analysis

GO term enrichment was determined by comparing the number of DEGs to the number of expressed genes in each GO term with Ontologizer [92] software: the term-for-term approach was used for overrepresentation statistical analysis and a Bonferroni correction for multiple testing was applied. Maize GO annotation was retrieved from maize-GAMER project [93]. The functional analysis of differential expression genes (DEGs) was performed using MapMan [94,95]: the overrepresentation of categories was determined using Fisher’s exact test and resulting p-values were adjusted according to Benjamini and Hochberg [96]. A critical cut-off value of 0.05 (corresponding to a Z-score ≥ 1.96) was applied to select enriched functional categories.

### 4.5. Maize Seedlings Growth Analysis

Roots and leaves from every treatment (-N or +NO_3_^−^ or +NH_4_^+^, 20 plants each) were separately sampled at T1 and T7, in three independent biological repetitions, and fresh weights were obtained. At T1, only a small first leaf was visible, while at T7 the leaf development reached the third leaf. Root and leaves images were collected using a flatbed scanner. Roots and leaves area and the primary root (PR) length were measured by means of ImageJ software analysis. Data represent the average of three independent biological replicates, each replicate considering 20 plants for every treatment (n = 20) ± standard error. For statistical analysis, data derived from the NO_3_^−^ or NH_4_^+^ treatment were compared with those of control plants (-N) using ANOVA test; data was considered significant when p < 0.05.

### 4.6. Chlorophyll, Flavonoids and Anthocyanins Optical Measurements

Thanks to the fluorescence’s properties of chlorophyll (CHL) and the characteristic of UV-A absorber of flavonoids (FLAV) and green light absorber of anthocyanins (ANTH), maize seedlings were evaluated for their epidermis absorbance in leaves using the optical sensor DUALEX SCIENTIFIC+^TM^ (Force-A, Orsay, France). In addition, the Nitrogen Balance Index (NBI) was directly obtained by the instrument as the ratio between CHL and FLAV content. At every time point (T3, T6 and T7), two lectures were performed on the first young leaf entirely developed for each plant in every treatment (-N or +NO_3_^−^ or +NH_4_^+^, 20 plants each). To assess FLAV content, readings were made on both adaxial and abaxial sides of leaves and them values were summed-up [97]. Three biological replicates for each treatment and an ANOVA statistic test were performed, each replicate considering 20 plants for every treatment (n = 20). Data represent the average of three replicates ± standard error.

### 4.7. Analysis of Amino Acids Using Hydrophilic Interaction Chromatography-Tandem Mass Spectrometry (HILIC-MS/MS)

Roots and leaves from every treatment (-N, +NO_3_^−^ and +NH_4_^+^, 20 plants each) were separately sampled at T7, in three independent biological repetitions, weight and immediately frozen in liquid nitrogen. Each sample was ground to fine powder in liquid nitrogen and exactly weighted (100 mg). Samples were then extracted in an ultrasound bath with a solution of diluted HCl (0.5 M) for 10 min at room temperature. Standard solutions were prepared, weighing the exact amount of each amino acid in diluted HCl solution (0.5 M) in four different concentrations from 10 µg/mL to 1 µg/mL. The solutions were centrifuged and used for analysis. For hydrolysis, samples were added of 15% Trichloroacetic acid (10 mL) and left at 80 °C for one night. pH of solution was then adjusted with ammonia solution (33%) to 2.0 and solutions were used for analysis. For analysis, an Agilent Z-HILIC Column was used as stationary phase (3 x 100 mm, 4 micron), eluents were acetonitrile, (A) and water 0.5% formic acid (B). The gradient started with 1 min to 95%A, then in 11 min to 70%A, then 14 min to 40%A then at 14.5 min back to 95%A. Flow rate was 0.450 mL/min. Each amino acid transition was optimized with corresponding standard solution. The transitions are reported in Table S9. The data represent the average of three independent biological replicates, each replicate considering 20 plants for every treatment (n = 20) ± standard error (SE). For statistical analysis, data derived from ANOVA test and are considered significantly different with *p* < 0.05.

## 5. Conclusions

In conclusion, this study provides a detailed picture of the global transcriptomic response of maize young roots to early nitrogen starvation or nitrate and ammonium supply, allowing us to distinguish common and individual elements of the overall response. In particular, different regulatory elements contributing to modulating the plant development and overall response to N availability were identified, thus providing a number of molecular targets which could be the starting point for further research in root biology or application in plant genetics and biotechnology to improve the Nitrogen Use Efficiency.

Furthermore, the present results also suggest that nitrate and ammonium could trigger the production of different secondary metabolites and the activation of unique hormonal pathways, thus affecting the tolerance to pathogens and abiotic stresses. In this perspective, the precise knowledge of these mechanisms could represent useful information for fine-tuning the nitrogen fertilization to improve resistance to stresses.

## Supplementary Materials

Supplementary materials can be found at https://www.mdpi.com/1422-0067/21/2/686/s1.

## Abbreviations

ANTH	Anthocyanins
CHL	Chlorophyll
DEG	Differentially Expressed Gene
FDR	False Discovery Rate
FLAV	Flavonoids
GO	Gene Ontology
HILIC-MS/MS	Hydrophilic interaction chromatography-Tandem Mass Spectrometry
LR	Lateral Root
NBI	Nitrogen Balance Index
NUE	Nitrogen Use Efficiency
PR	Primary Root
ROS	Reactive Oxygen Species
SLs	Strigolactones
