# Supplementary material for: Nitrate and Ammonium Affect the Overall Maize Response to Nitrogen Availability by Triggering Specific and Common Transcriptional Signatures in Roots
Source: Int J Mol Sci. 2020 Jan 20;21(2):686. doi: 10.3390/ijms21020686 (PMC7013554; doi:10.3390/ijms21020686)

Figure S1: Percentage of differentially expressed genes (DEGs) identified (log2 FC >|0.58|; FDR≤0.05) by RNA-Seq analysis from the comparison between NO_3^- or NH_4^+ supplied maize seedlings for 24 h with respect to the control (-N, nitrogen deficient solution). Data are shown as percentage of genes differentially expressed in response to each treatment in +NO_3^- (A) and +NH_4^+ (B) treatments on the total amount of DEGs. DEGs were classified as up-regulated according to their log2 fold change values (a log2FC threshold > |0.58| was set, corresponding to a 1.5-fold change increase or decrease in expression). Among the up- and downregulated DEGs, several ranges of induction or repression are shown as log2 of the gene expression fold changes.

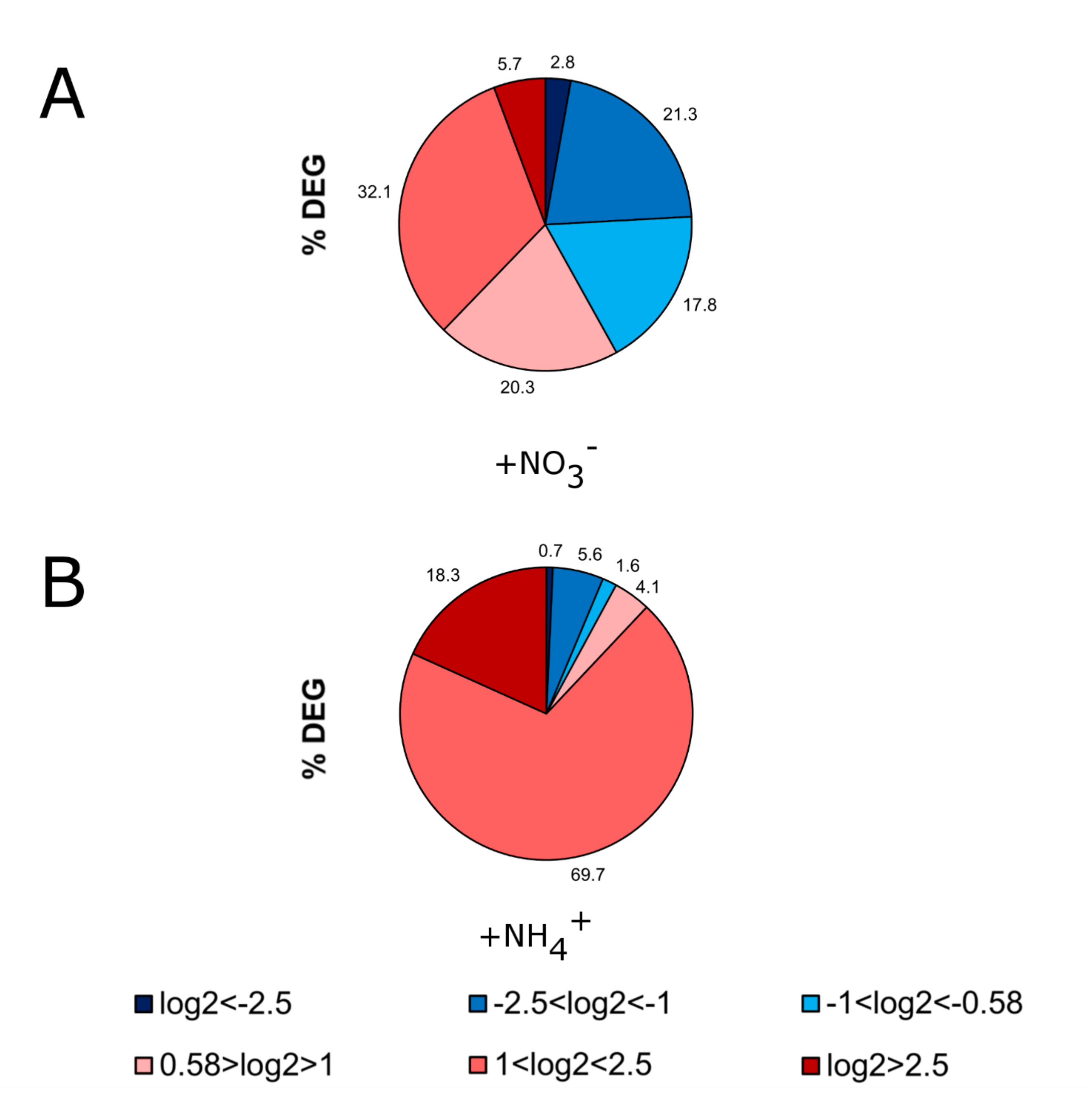

Supplement: Supplementary file 1 [file ijms-21-00686-s001.zip › SUPPLEMENTARY MATERIALS_Ravazzolo_et_al_2019_IJMS/Figure S1.pdf]
