# Supplementary material for: Nitrate and Ammonium Affect the Overall Maize Response to Nitrogen Availability by Triggering Specific and Common Transcriptional Signatures in Roots
Source: Int J Mol Sci. 2020 Jan 20;21(2):686. doi: 10.3390/ijms21020686 (PMC7013554; doi:10.3390/ijms21020686)
Supplement: Supplementary file 1 [file ijms-21-00686-s001.zip › SUPPLEMENTARY MATERIALS_Ravazzolo_et_al_2019_IJMS/suppl information caption_Ravazzolo.pdf]

Nitrate and ammonium affect the overall maize response to nitrogen availability by triggering specific and common transcriptional signatures in root

Laura Ravazzolo[1], Sara Trevisan[1], Cristian Forestan[1], Serena Varotto[1], Stefania Sut[1], Stefano Dall'Acqua[2], Mario Malagoli[1], and Silvia Quaggiotti[1*]

[1] Dept. of Agronomy, Food, Natural resources, Animals and Environment, University of Padova, Agripolis – V.le dell'Università, 16, 35020 Legnaro (PD), Italy; laura.ravazzolo@unipd.it; sara.trevisan@unipd.it; cristian.forestan@unipd.it; serena.varotto@unipd.it; mario.malagoli@unipd.it

[2] Dept. of Pharmaceutical and Pharmacological Sciences, University of Padova - Via Marzolo 5, 35121 Padova, Italy; stefania.sut@phd.unipd.it; stefano.dallacqua@unipd.it

* Correspondence: silvia.quaggiotti@unipd.it; Tel.: +39 049 8272913

Supplementary information

Figure S1: Distribution and percentage of differentially expressed genes (DEGs) identified (log2 FC >|0.58|; FDR≤0.05) by RNA-Seq analysis from the comparison between NO_3^- or NH_4^+ supplied maize seedlings for 24 h with respect to the control (-N, nitrogen deficient solution). Data are shown as percentage of genes differentially expressed in response to each treatment in $+NO_3^-$ treatment (A) and $+NH_4^+$ (B) on the total amount of DEGs. DEGs were classified as up-regulated according to their log2 fold change values (a log2FC threshold > |0.58| was set, corresponding to a 1.5-fold change increase or decrease in expression). Among the up- and downregulated DEGs, several ranges of induction or repression are shown as log2 of the gene expression fold changes.

Supplementary dataset 1: Cuffdiff complete results of pairwise differential expression analyses ($+NO_3^-$ vs -N; $+NH_4^+$ vs -N).

Supplementary dataset 2: Complete results of GO enrichment analysis for the DEGs included in each cluster.

Table S1: Summary of reads obtained by RNA-Seq analysis. For each treatment ($+NO_3^-$, $+NH_4^+$) or control condition (-N), three biological replicates were processed (R1, R2, R3).

Table S2: Expression matrix of differentially expressed genes (DEGs) showing a log2 fold change ratio >|0.58| and a false discovery rate (FDR)-adjusted p-value ≤ 0.05 in at least one treatment that were included in the hierarchical clustering analysis. RPMK expression values in $+NO_3^-$, -N and $+NH_4^+$ samples and clustering results are reported for each of the 2324 DEGs.

Table S3: List of some selected differentially expressed genes (DEGs) showing a log2 fold change ratio >|0.58| and a false discovery rate (FDR)-adjusted p-value ≤ 0.05 in at least one treatment that are related to N transport and metabolism. RPMK (Reads Per Kb per Million) expression values in

+NO_3^-, -N and +NH_4^+ samples, hierarchical clustering results and log2 fold change ratio are reported.

Table S4: List of representative genes from selected GO terms overrepresented among DEGs specifically responsive to NH_4^+. RPKM: Reads Per Kb per Million.

Table S5: List of representative genes from selected GO terms overrepresented among DEGs specifically responsive to NO_3^-. RPKM: Reads Per Kb per Million.

Table S6: List of representative genes from selected GO terms overrepresented among DEGs specifically responsive to both NH_4^+ and NO_3^-. RPKM: Reads Per Kb per Million.

Table S7: Complete quantification for all free amino acids detected as average of μg/g of weighted tissue ± SE and the proportion of total amino acids (%). TOT: calculated sum of total amino acids. Δ% -N: calculated percentage increase/decrease of total free amino acids detected in each treatment (+NO_3^- or +NH_4^+) with respect to the N-deficient treatment (-N). The asterisk indicates significant differences with respect to -N treatments (Student's t-test with: ** $p \leq 0.01$, * $p \leq 0.05$).

Table S8: Complete quantification for all hydrolysed amino acids detected as average of μg/g of weighted tissue ± SE and the proportion of total amino acids (%). TOT: calculated sum of total amino acids. Δ% -N: calculated percentage increase/decrease of total hydrolysed amino acids detected in each treatment (+NO_3^- or +NH_4^+) with respect to the N-deficient treatment (-N). The asterisk indicates significant differences with respect to -N treatments (Student's t-test with: ** $p \leq 0.01$, * $p \leq 0.05$).

Table S9: Amino acid transitions for HILIC-MS/MS quantification.
