# Supplementary material for: Nitrate and Ammonium Affect the Overall Maize Response to Nitrogen Availability by Triggering Specific and Common Transcriptional Signatures in Roots
Source: Int J Mol Sci. 2020 Jan 20;21(2):686. doi: 10.3390/ijms21020686 (PMC7013554; doi:10.3390/ijms21020686)
Supplement: Supplementary file 1 [file ijms-21-00686-s001.zip › SUPPLEMENTARY MATERIALS_Ravazzolo_et_al_2019_IJMS/Table S1.pdf]

Table S1. Summary of reads obtained by RNA-Seq analysis. For each treatment (+NO_3^-, +NH_4^+) or control condition (-N), three biological replicates were processed (R1, R2, R3).

**Result**	**-N libraries**					
	R1		R2		R3	
N° of total reads	26357768		27157883		30178515	
N° of high-quality reads	26254402	99.61%	27051232	99.61%	30045802	99.56%
N° of mapped reads	25805014	98.29%	26614982	98.39%	29474225	98.10%
Uniquely mapped reads	23890148	92.58%	24711441	92.85%	26654323	90.43%
Multi-mapped reads	1914866	7.42%	1903541	7.15%	2819902	9.57%
Unmapped reads	103366	1.71%	106651	1.61%	132713	1.90%
**Result**	**+NO_3^- libraries**					
	R1		R2		R3	
N° of total reads	26061640		32597592		28820292	
N° of high-quality reads	25946454	99.56%	32459506	99.58%	28694329	99.56%
N° of mapped reads	25477079	98.19%	31900192	98.28%	28073982	97.84%
Uniquely mapped reads	23203264	91.08%	28856879	90.46%	24892495	88.67%
Multi-mapped reads	2273815	8.92%	3043313	9.54%	3181487	11.33%
Unmapped reads	115186	1.81%	138086	1.72%	125963	2.16%
**Result**	**+NH_4^+ libraries**					
	R1		R2		R3	
N° of total reads	35121464		25513916		23264596	
N° of high-quality reads	34934389	99.47%	25352921	99.37%	23145412	99.49%
N° of mapped reads	34296996	98.18%	24704601	97.44%	22609271	97.68%
Uniquely mapped reads	31201251	90.97%	22009628	89.09%	20703717	91.57%
Multi-mapped reads	3095745	9.03%	2694973	10.91%	1905554	8.43%
Unmapped reads	187075	1.82%	160995	2.56%	119184	2.32%
