# Supplementary material for: Nitrate and Ammonium Affect the Overall Maize Response to Nitrogen Availability by Triggering Specific and Common Transcriptional Signatures in Roots
Source: Int J Mol Sci. 2020 Jan 20;21(2):686. doi: 10.3390/ijms21020686 (PMC7013554; doi:10.3390/ijms21020686)
Supplement: Supplementary file 1 [file ijms-21-00686-s001.zip › SUPPLEMENTARY MATERIALS_Ravazzolo_et_al_2019_IJMS/Table S3.pdf]

Table S3: List of some selected differentially expressed genes (DEGs) showing a log2 fold change ratio >|0.58| and a false discovery rate (FDR)-adjusted p-value ≤ 0.05 in at least one treatment that are related to N transport and metabolism. RPMK (Reads Per Kb per Million) expression values in $+NO_3^-$, -N and $+NH_4^+$ samples, hierarchical clustering results and log2 fold change ratio are reported.

**Gene_description**	**Gene_id**	**RPKM**			**Cluster**	**log2 $+NO_3^-$/-N**	**log2 $+NH_4^+$/-N**
		**$+NO_3^-$**	**-N**	**$+NH_4^+$**			
Ammonium transporter 2	Zm00001d034782	2.94596	8.57231	8.49429	7	-1.54095	-0.01319
Ammonium transporter 2	Zm00001d016771	4.31905	3.89863	9.26185	1	0.147746	1.248333
ammonium transporter 1, ZmAMT1;1a	Zm00001d025831	3.91086	8.7152	19.4092	7	-1.15605	1.155135
Ammonium transporter 1	Zm00001d025831	3.91086	8.7152	19.4092	7	-1.15605	1.155135
Ammonium transporter 2	Zm00001d017249	0.644629	2.81132	3.23104	7	-2.12471	0.200751
Ammonium transporter 2	Zm00001d017249	0.644629	2.81132	3.23104	7	-2.12471	0.200751
Ferredoxin-6, chloroplastic Precursor (Ferredoxin VI) (Fd VI)	Zm00001d038291	37.1851	10.1148	34.5976	3	1.87826	1.774204
Ferredoxin--NADP reductase root isozyme 1 chloroplastic	Zm00001d034345	110.226	52.267	83.73	3	1.07649	0.679844
Ferredoxin--nitrite reductase chloroplastic	Zm00001d018161	16.3465	0.820326	16.7096	3	4.31664	4.348336
glucose-6-phosphate translocator	Zm00001d021653	3.54728	0.996285	1.10639	5	1.83208	0.15123
Glutamine synthetase leaf isozyme chloroplastic	Zm00001d026501	13.5914	4.93134	6.99464	5	1.46264	0.50427
Hemoglobin 1	Zm00001d048020	300.534	33.1356	228.802	3	3.18107	2.787646
Hemoglobin 2	Zm00001d038718	5.72224	0.313381	0.830336	5	4.19059	1.405778
NRa - nitrate reductase (NADH)1	Zm00001d049995	1.77204	0.291422	1.69453	3	2.60423	2.539703
Urea-proton symporter DUR3	Zm00001d037242	0.143906	0.666156	1.07956	7	-2.21073	0.696511
ZmNRT1a, nitrate transporter/peptide transporter family 2	Zm00001d029932	27.3563	17.7566	22.7131	3	0.623519	0.355169
ZmNRT1b, protein NRT1/ PTR FAMILY 4.6	Zm00001d036941	1.58251	1.28707	3.78195	1	0.298119	1.55504
