# Supplementary material for: Nitrate and Ammonium Affect the Overall Maize Response to Nitrogen Availability by Triggering Specific and Common Transcriptional Signatures in Roots
Source: Int J Mol Sci. 2020 Jan 20;21(2):686. doi: 10.3390/ijms21020686 (PMC7013554; doi:10.3390/ijms21020686)
Supplement: Supplementary file 1 [file ijms-21-00686-s001.zip › SUPPLEMENTARY MATERIALS_Ravazzolo_et_al_2019_IJMS/Table S4_S5_S6.pdf]

Table S4: List of representative genes from selected GO terms overrepresented among DEGs specifically responsive to NH_4^+. RPKM: Reads Per Kb per Million.

CLUSTER 1: genes up-regulated by NH_4^+						
GO ID	GO enriched term	Gene ID	Gene Description	RPKM $+NO_3^-$	RPKM -N	RPKM $+NH_4^+$
GO:0009725	*response to hormone*	Zm00001d011208	1-aminocyclopropane-1-carboxylate oxidase 1	187.99	115.85	438.95
		Zm00001d019078	AAA-ATPase ASD mitochondrial	28.80	12.73	104.28
		Zm00001d052493	Aluminium induced protein with YGL and LRDR motifs	194.17	130.31	438.88
		Zm00001d022581	AMP deaminase	66.38	61.58	154.14
		Zm00001d015091	Bax inhibitor 1	124.55	83.38	314.62
		Zm00001d044389	calcineurin B-like5	22.54	25.71	144.21
		Zm00001d002540	calcium dependent protein kinase5	65.34	51.94	103.99
		Zm00001d044516	DCD (Development and Cell Death) domain protein	79.97	76.84	146.37
		Zm00001d038999	drought-induced 19	301.31	185.01	517.20
		Zm00001d028966	ELMO/CED-12 family protein	70.13	69.56	155.94
		Zm00001d022461	ERF-like protein	13.31	10.25	170.67
		Zm00001d040651	Ethylene-responsive transcription factor RAP2-2	22.68	17.08	107.85
		Zm00001d042096	Glutathione S-transferase U16	66.46	50.87	110.59
		Zm00001d027557	glutathione transferase31	13.16	10.50	123.40
		Zm00001d034356	glutathione transferase5	88.58	79.89	188.49
		Zm00001d018809	glutathione transferase6	56.27	41.15	126.00
		Zm00001d024778	Hydrophobic protein RCI2B	403.78	285.45	1045.20
		Zm00001d018973	IAA24-auxin-responsive Aux/IAA family member	30.90	27.88	87.24
		Zm00001d045298	Lipase-like	56.03	54.46	98.77
		Zm00001d011638	Lipid phosphate phosphatase 2	14.77	34.87	101.74
		Zm00001d049954	Monodehydroascorbate reductase 1 peroxisomal	84.94	105.90	220.10
		Zm00001d019207	NAC domain-containing protein 2	14.46	14.70	83.70

		Zm00001d014608	Peroxidase 16	40.41	20.49	117.40
		Zm00001d010478	Peroxisomal adenine nucleotide carrier 1	33.27	32.48	99.53
		Zm00001d007037	Probable mediator of RNA polymerase II transcription subunit 26b	25.82	21.64	74.64
		Zm00001d018668	Protein C2-DOMAIN ABA-RELATED 4	173.47	120.10	265.33
		Zm00001d005193	Protein LURP1	44.85	38.38	83.92
		Zm00001d021413	Protein PIN-LIKES 7	60.88	47.64	180.18
		Zm00001d022139	Protein TIFY 10B	164.63	83.96	300.88
		Zm00001d006860	Protein TIFY 10B	46.74	36.28	86.72
		Zm00001d009103	Putative AP2/EREBP transcription factor superfamily protein	108.19	79.84	474.03
		Zm00001d025081	Rhodanese-like domain-containing protein 19 mitochondrial	150.25	111.85	353.51
		Zm00001d013005	RING/U-box superfamily protein	42.20	38.90	87.26
		Zm00001d039407	Shaggy-related protein kinase iota	38.36	38.59	85.28
		Zm00001d039052	Sugar transporter ERD6-like 6	67.68	55.00	120.20
		Zm00001d041716	Syntaxin-132	24.45	30.85	88.42
GO:0009414	*response to water deprivation*	Zm00001d017762	abscisic acid 8'-hydroxylase 1	16.20	16.13	32.60
		Zm00001d004731	Aldehyde dehydrogenase	19.09	25.93	61.10
		Zm00001d026191	AP2-EREBP transcription factor	2.39	1.54	5.40
		Zm00001d044285	calcineurin B-like 10	43.97	30.00	65.20
		Zm00001d044389	calcineurin B-like 5	22.54	25.71	144.21
		Zm00001d002540	calcium dependent protein kinase 5	65.34	51.94	103.99
		Zm00001d007097	Early response to dehydration 15-like protein	10.16	4.13	25.72
		Zm00001d027557	glutathione S-transferase subunit 31	13.16	10.50	123.40
		Zm00001d034356	glutathione transferase 5	88.58	79.89	188.49
		Zm00001d018809	glutathione transferase 6	56.27	41.15	126.00
		Zm00001d008795	Glycosyltransferase	4.32	3.54	12.85
		Zm00001d021168	Glycosyltransferase	0.24	0.66	13.55
		Zm00001d020714	HSF transcription factor	0.79	0.51	5.01
		Zm00001d006016	Multiple stress-responsive zinc-finger protein ISAP1	9.48	4.37	31.42
		Zm00001d042609	NAC domain-containing protein 48	19.94	15.22	45.13
		Zm00001d024778	NaCl stress protein 1	403.78	285.45	1045.20

		Zm00001d036879	Non-specific serine/threonine protein kinase	14.38	19.12	37.30
		Zm00001d032155	Non-specific serine/threonine protein kinase	4.91	3.01	12.15
		Zm00001d010478	Peroxisomal carrier protein	33.27	32.48	99.53
		Zm00001d029397	phospholipase D7	1.73	4.81	20.23
		Zm00001d010399	Putative WRKY DNA-binding domain superfamily protein	12.08	9.43	28.99
		Zm00001d049173	Putative WRKY DNA-binding domain superfamily protein	0.13	0.14	3.60
		Zm00001d025545	Zeaxanthin epoxidase, chloroplastic	7.00	7.66	21.20
GO:0009607	*response to biotic stimulus*	Zm00001d011208	1-aminocyclopropane-1-carboxylate oxidase 1	187.99	115.85	438.95
		Zm00001d024843	1-aminocyclopropane-1-carboxylate oxidase15	1.23	0.79	3.09
		Zm00001d024853	1-aminocyclopropane-1-carboxylate oxidase15	2.97	4.31	13.16
		Zm00001d017762	abscisic acid 8'-hydroxylase1	16.20	16.13	32.56
		Zm00001d047942	Actin-depolymerizing factor 5	28.90	20.46	43.68
		Zm00001d014692	AIG2-like protein	22.09	17.91	37.28
		Zm00001d052485	AMP-binding protein	9.62	8.50	23.88
		Zm00001d026271	AP2/EREBP putative transcription factor superfamily protein	0.03	0.00	0.73
		Zm00001d026191	AP2-EREBP transcription factor	2.39	1.54	5.39
		Zm00001d037941	AP2-EREBP transcription factor	30.89	22.62	61.48
		Zm00001d016456	ARID transcription factor	4.41	3.19	9.51
		Zm00001d017912	ASC1-like protein 2	32.46	40.76	84.04
		Zm00001d033981	ATP sulfurylase1	12.96	11.50	29.84
		Zm00001d044095	Carbonic anhydrase	2.80	1.54	6.28
		Zm00001d025753	chitinase B1	1.12	0.27	2.83
		Zm00001d053432	Cytochrome b6-f complex iron-sulfur subunit	0.59	0.92	13.27
		Zm00001d012322	Cytochrome P450 CYP81A1	0.12	0.41	9.41
		Zm00001d009666	Dihydroflavonol-4-reductase	3.52	4.24	29.04
		Zm00001d007097	Early response to dehydration 15-like protein	10.16	4.13	25.72
		Zm00001d033906	Endoplasmic oxidoreductin-1	21.63	26.39	70.87
		Zm00001d035854	Fatty acid alpha-dioxygenase	0.60	2.97	39.62
		Zm00001d023830	Fiber protein Fb19	46.74	41.32	113.20
		Zm00001d019277	Flower-specific gamma-thionin	6.58	4.45	17.84

		Zm00001d049926	Gibberellin 20 oxidase 2	0.26	0.14	7.49
		Zm00001d039642	Glycosyltransferase	1.54	3.98	20.37
		Zm00001d043166	Glycosyltransferase	2.91	2.16	5.80
		Zm00001d020714	HSF transcription factor	0.79	0.51	5.01
		Zm00001d036073	Hypersensitive-induced reaction protein 4	28.55	31.72	60.24
		Zm00001d021843	Low-molecular-weight cysteine-rich protein LCR70	11.91	7.86	114.95
		Zm00001d040468	major facilitator superfamily defense 1 related to integral membrane permeases	1.92	0.72	3.94
		Zm00001d010271	MED26 transcription factor	18.58	19.10	49.63
		Zm00001d022314	MED26 transcription factor	2.95	2.20	9.92
		Zm00001d042609	NAC domain-containing protein 48	19.94	15.22	45.13
		Zm00001d012527	NAC transcription factor	7.70	4.04	52.29
		Zm00001d017025	Nitrate reductase	0.22	0.11	1.15
		Zm00001d028733	Non-specific serine/threonine protein kinase	14.88	17.87	39.01
		Zm00001d026649	opaque endosperm7: like o1; high lysine content	8.60	7.65	22.46
		Zm00001d031267	Oxidoreductase	28.45	32.59	148.89
		Zm00001d028815	Pathogenesis-related protein 10	36.25	24.66	83.56
		Zm00001d032992	Pectinesterase	30.66	35.19	83.31
		Zm00001d010218	Peroxidase	0.15	0.24	9.60
		Zm00001d014341	Peroxidase	2.50	2.20	6.14
		Zm00001d010478	Peroxisomal carrier protein	33.27	32.48	99.53
		Zm00001d007765	Prolyl 4-hydroxylase 4	24.98	13.05	80.74
		Zm00001d018526	Prolyl 4-hydroxylase 8	27.89	29.78	63.76
		Zm00001d005018	Protein DETOXIFICATION	10.85	8.30	28.30
		Zm00001d015964	Protein DETOXIFICATION	3.48	2.78	9.34
		Zm00001d053087	Putative D-mannose binding lectin receptor-like protein kinase family protein	4.11	2.50	9.03
		Zm00001d023669	Putative NAC domain transcription factor superfamily protein	11.45	10.36	26.46
		Zm00001d046261	Putative thaumatin domain family protein	1.64	1.42	7.40
		Zm00001d050164	Putative WAK-related receptor-like protein kinase family protein	0.66	0.25	1.47
		Zm00001d010399	Putative WRKY DNA-binding domain superfamily protein	12.08	9.43	28.99
		Zm00001d010617	Putative WRKY DNA-binding domain superfamily protein	5.11	4.03	15.53

GO ID	GO enriched term	Gene ID	Gene Description	RPKM $+NO_3^-$	RPKM -N	RPKM $+NH_4^+$
		Zm00001d008651	Pyruvate decarboxylase	2.14	4.90	53.34
		Zm00001d028759	Pyruvate decarboxylase	230.40	224.75	589.37
		Zm00001d010588	pyruvate decarboxylase1	105.13	73.74	274.49
		Zm00001d052653	respiratory burst oxidase4	7.61	6.70	23.15
		Zm00001d012221	stearoyl-acyl-carrier-protein desaturase9	47.49	41.20	124.84
		Zm00001d035066	STIP1 homology and U box-containing protein 1	12.35	10.80	24.79
		Zm00001d009990	Superoxide dismutase	167.60	183.97	346.11
		Zm00001d022139	TIFY27	164.63	83.96	300.88
		Zm00001d007357	Transport inhibitor response 1 protein	19.73	16.28	35.10
		Zm00001d018969	tryptophan synthase alpha subunit1	26.28	27.77	54.56
		Zm00001d002896	TUB-transcription factor 4	4.39	4.77	16.11
		Zm00001d009118	Universal stress protein	113.53	77.27	699.62
		Zm00001d052776	VAMP protein SEC22	2.86	2.13	8.13
		Zm00001d053746	WRKY71-superfamily of TFs having WRKY and zinc finger domains	2.85	1.51	10.55
		Zm00001d020136	WRKY74-superfamily of TFs having WRKY and zinc finger domains	6.21	3.08	11.11
		Zm00001d042055	Zea mays ARGOS6	16.03	11.31	37.84
**CLUSTER 6: genes down-regulated by NH_4^+**						
GO ID	GO enriched term	Gene ID	Gene Description	RPKM $+NO_3^-$	RPKM -N	RPKM $+NH_4^+$
GO:0008283	*cell proliferation*	Zm00001d012166	Faciata 1-like protein	6.89	8.13	3.52
		Zm00001d018415	Proliferating cell nuclear antigen	72.96	78.34	39.10

Table S5: List of representative genes from selected GO terms overrepresented among DEGs specifically responsive to NO_3^-. RPKM: Reads Per Kb per Million.

CLUSTER 5: genes up-regulated by NO_3^-						
GO ID	GO enriched term	Gene ID	Gene Description	RPKM $+NO_3^-$	RPKM -N	RPKM $+NH_4^+$
GO:0048046	*apoplast*	Zm00001d004443	Mannitol dehydrogenase	2.80	0.97	0.58
		Zm00001d005472	Dirigent protein	44.75	25.31	26.45
		Zm00001d038049	Lichenase-2	19.76	10.44	13.01
		Zm00001d046357	Beta-galactosidase	52.40	32.83	24.62
		Zm00001d047775	Dirigent protein	19.55	8.08	12.20
		Zm00001d048669	Auxin-induced beta-glucosidase	9.67	5.14	4.82
		Zm00001d052336	Peroxidase	60.22	39.95	38.00
		Zm00001d052585	Dirigent protein	46.70	1.53	1.45
		Zm00001d053585	Beta-galactosidase	7.21	4.36	5.22
GO:0030247	*polysaccharide binding*	Zm00001d038049	Lichenase-2	19.76	10.4	13.0
		Zm00001d043361	Glucan endo-1,3-beta-glucosidase 1	38.72	24.2	17.2
**CLUSTER 7: genes down-regulated by NO_3^-**						
GO ID	GO enriched term	Gene ID	Gene Description	RPKM $+NO_3^-$	RPKM -N	RPKM $+NH_4^+$
GO:0044550	*secondary metabolite biosynthetic process*	Zm00001d017077	red aleurone1: changes purple aleurone to red; encodes flavonoid 3'-hydroxylase	4.71	10.59	8.57
		Zm00001d017279	phenylalanine ammonia lyase7	2.71	6.61	7.49
		Zm00001d018791	Anther-specific proline-rich protein APG	0.64	2.03	1.60
		Zm00001d023258	Calcium-transporting ATPase	9.10	13.72	16.97
		Zm00001d024763	3-N-debenzoyl-2-deoxytaxol N-benzoyltransferase	75.08	142.23	137.22
		Zm00001d035115	multidrug and toxic compound extrusion1: confers aluminum tolerance	14.06	49.09	129.60
		Zm00001d045043	Mannitol dehydrogenase	28.51	50.39	72.22
		Zm00001d046493	heme oxygenase3: encodes a heme oxygenase	5.81	12.56	13.16

		Zm00001d048814	Dirigent protein	1.21	4.33	3.30
GO:0098655	*cation transmembrane transport*	Zm00001d034782	Ammonium transporter	2.95	8.57	8.49
		Zm00001d025831	ammonium transporter1	3.91	8.72	19.41
		Zm00001d017249	ammonium transporter2	0.64	2.81	3.23
		Zm00001d023258	Calcium-transporting ATPase	9.10	13.72	16.97
		Zm00001d018799	Non-specific serine/threonine protein kinase	69.05	109.51	186.54
		Zm00001d002006	Plasma membrane ATPase	271.85	506.21	718.06
		Zm00001d003861	Potassium transporter	21.24	57.80	55.67
		Zm00001d020325	Potassium transporter	32.55	49.22	43.96
		Zm00001d022485	Potassium transporter	6.02	10.60	14.65

Table S6: List of representative genes from selected GO terms overrepresented among DEGs specifically responsive to both NH_4^+ and NO_3^-. RPKM: Reads Per Kb per Million.

CLUSTER 2;3: genes up-regulated by both NO_3^- and NH_4^+						
GO ID	GO enriched term	Gene ID	Gene Description	RPKM $+NO_3^-$	RPKM -N	RPKM $+NH_4^+$
GO:0009725	*response to hormone*	Zm00001d006119	2Fe-2S ferredoxin-like superfamily protein	10.91	3.15	6.39
		Zm00001d023779	3-hydroxyisobutyryl-CoA hydrolase 1	42.60	26.62	42.44
		Zm00001d003209	AAA-ATPase ASD mitochondrial	7.31	3.62	9.91
		Zm00001d053396	Abscisic acid receptor PYL9	12.65	7.08	13.44
		Zm00001d033167	ACT domain-containing protein ACR4	20.05	11.39	25.19
		Zm00001d025047	APx3-Peroxisomal Ascorbate Peroxidase	15.78	7.62	21.16
		Zm00001d029475	ATP-dependent protease La (LON) domain protein	3.07	1.14	3.11
		Zm00001d041056	Auxin response factor 2	2.11	0.53	1.30
		Zm00001d033711	Basic blue protein	23.22	10.14	16.24
		Zm00001d053952	Bax inhibitor 1	27.13	15.56	35.33
		Zm00001d007718	benzoxazinone synthesis13	54.64	12.39	55.50
		Zm00001d043389	CBL-interacting serine/threonine-protein kinase 18	19.80	12.38	23.69
		Zm00001d048460	CBL-interacting serine/threonine-protein kinase 9	8.42	5.23	11.88
		Zm00001d017547	Dehydrin COR410	17.32	3.89	11.54
		Zm00001d034160	DNA binding protein	38.28	25.24	50.37
		Zm00001d029102	Dormancy-associated protein homolog 3	20.57	12.35	20.50
		Zm00001d032295	DRE-binding protein 1	8.55	2.23	8.50
		Zm00001d021141	Ferredoxin	13.59	4.18	9.06
		Zm00001d034345	Ferredoxin--NADP reductase root isozyme 1 chloroplastic	110.23	52.27	83.73
		Zm00001d019985	GDSL esterase/lipase	5.80	2.88	4.60
		Zm00001d013222	Gibberellin-regulated protein 10	40.06	25.49	43.96
		Zm00001d048354	Glutathione S-transferase F9	27.14	17.01	29.42

		Zm00001d043795	Glutathione S-transferase GSTU6	52.62	27.30	53.63
		Zm00001d029708	glutathione transferase30	5.08	1.38	4.91
		Zm00001d042104	glutathione transferase7	6.09	3.05	8.34
		Zm00001d046472	Hemoglobin-like protein HbO	39.46	27.79	54.05
		Zm00001d048950	Hevein-like preproprotein	4.14	1.33	4.08
		Zm00001d005867	HVA22-like protein d	6.48	2.62	5.91
		Zm00001d011753	HVA22-like protein f	6.64	2.39	8.40
		Zm00001d024546	late hypocotyl elongation protein ortholog1: myb transcription factor-core clock gene	4.93	2.67	3.90
		Zm00001d035756	Leucine-rich repeat (LRR) family protein	6.14	1.35	10.20
		Zm00001d004706	Lipase-like PAD4	3.91	1.50	3.00
		Zm00001d041204	lipoxygenase12	20.01	7.24	17.55
		Zm00001d029506	LOB domain-containing protein 42	8.39	3.84	11.74
		Zm00001d029963	myb-like transcription factor family protein	31.60	17.83	41.44
		Zm00001d047441	NADPH:quinone oxidoreductase	72.92	29.46	98.79
		Zm00001d029529	Ninja-family protein AFP3	25.40	12.83	20.14
		Zm00001d048947	pathogenesis-related protein3	15.88	4.25	13.19
		Zm00001d027727	Phosphoserine aminotransferase 1 chloroplastic	67.74	43.64	86.69
		Zm00001d038741	Pistil-specific extensin-like protein	13.33	2.19	10.22
		Zm00001d018857	Probable carboxylesterase 15	6.27	1.60	6.93
		Zm00001d010284	Probable carboxylesterase 17	7.27	2.51	4.74
		Zm00001d038178	Probable carboxylesterase 17	2.78	0.66	1.63
		Zm00001d001774	Probable CCR4-associated factor 1 homolog 11	2.20	0.39	3.43
		Zm00001d009700	Probable desiccation-related protein LEA14	73.87	44.62	76.93
		Zm00001d022122	Probable GTP diphosphokinase RSH2 chloroplastic	2.02	0.97	1.67
		Zm00001d002564	Protein DMR6-LIKE OXYGENASE 2	1.59	0.30	1.26
		Zm00001d029932	Protein NRT1/ PTR FAMILY 6.3	27.36	17.76	22.71
		Zm00001d016119	Protein SOMBRERO	4.77	1.17	2.84
		Zm00001d005813	Protein TIFY 10B	1.58	0.32	0.85
		Zm00001d027900	Protein TIFY 10B	2.24	0.63	4.33
		Zm00001d026139	Putative calcium-dependent protein kinase family protein	4.97	2.28	6.15

		Zm00001d038300	Putative cytochrome P450 superfamily protein	5.01	2.23	8.17
		Zm00001d051891	Putative LOB domain-containing family protein	4.96	1.99	3.21
		Zm00001d023402	Putative MYB DNA-binding domain superfamily protein	1.68	0.41	1.32
		Zm00001d017964	Putative RNA-dependent RNA polymerase	3.20	1.83	3.12
		Zm00001d038843	Putative WRKY DNA-binding domain superfamily protein	6.15	2.40	10.05
		Zm00001d017786	putidaredoxin reductase homolog1	3.94	1.95	6.45
		Zm00001d022628	R2R3MYB-domain protein	42.41	25.92	36.09
		Zm00001d021839	Remorin family protein	35.06	21.92	44.55
		Zm00001d040445	RING/U-box superfamily protein	10.74	5.08	8.22
		Zm00001d032550	RNA-binding protein	8.92	4.56	11.79
		Zm00001d010751	Seed specific protein Bn15D17A	6.44	2.17	5.52
		Zm00001d038717	Seed specific protein Bn15D17A	7.71	3.31	9.23
		Zm00001d026632	Stem-specific protein TSJT1	38.64	11.95	56.70
		Zm00001d035683	Subtilisin-chymotrypsin inhibitor CI-1	318.75	198.12	455.65
		Zm00001d023677	sugars will eventually be exported transporter13a	23.40	14.00	18.24
		Zm00001d050577	sugars will eventually be exported transporter15a	1.90	0.21	0.96
		Zm00001d014793	thick tassel dwarf1	1.70	0.75	1.33
		Zm00001d022277	tolB protein-related	3.40	1.37	5.21
		Zm00001d011778	tonoplast intrinsic protein1	8.91	2.80	8.01
		Zm00001d052254	Transcription factor PRE3	6.64	2.72	7.45
		Zm00001d042463	Two-component response regulator ARR11	14.91	7.33	12.50
		Zm00001d044106	UDP-glycosyltransferase 73B3	1.58	0.35	2.93
		Zm00001d033980	ustilago maydis induced12	6.86	3.19	11.28
		Zm00001d013705	VAN3-binding protein	3.14	1.40	2.54
		Zm00001d018195	WAT1-related protein	19.85	9.48	28.10
		Zm00001d033284	WAT1-related protein	12.09	6.03	9.75
		Zm00001d036440	WAT1-related protein	19.44	9.44	17.29
		Zm00001d009309	Xyloglucan endotransglucosylase/hydrolase protein 14	12.81	5.75	10.56
		Zm00001d014613	Xyloglucan endotransglucosylase/hydrolase protein 23	22.64	12.36	29.13

CLUSTER 8: genes down-regulated by both NO_3^- and NH_4^+						
GO ID	GO enriched term	Gene ID	Gene Description	RPKM $+NO_3^-$	RPKM -N	RPKM $+NH_4^+$
GO:0005200	*structural constituent of cytoskeleton*	Zm00001d040509	beta tubulin5	166.29	365.37	230.50
		Zm00001d006651	Tubulin alpha chain	164.52	276.36	148.24
		Zm00001d021891	Tubulin alpha chain	392.24	642.07	403.73
		Zm00001d008216	Tubulin beta chain	37.58	57.19	36.44
GO:0072593	*reactive oxygen species metabolic process*	Zm00001d033751	Dynamin-related protein 1C	43.50	65.80	38.33
		Zm00001d040364	Peroxidase	424.76	889.51	498.52
		Zm00001d038598	Peroxidase	55.45	136.74	70.91
		Zm00001d022283	Peroxidase	6.84	62.69	27.63
		Zm00001d050572	Peroxidase	22.99	59.47	35.58
		Zm00001d022280	Peroxidase	5.50	39.74	19.56
		Zm00001d010924	Peroxidase	17.32	29.21	23.21
		Zm00001d045845	Peroxidase	6.19	15.92	10.62
		Zm00001d045846	Peroxidase	3.17	15.71	7.25
		Zm00001d022281	Peroxidase	3.04	9.54	6.07
		Zm00001d028348	Peroxidase	0.28	3.67	1.29
		Zm00001d026357	Peroxidase	0.15	1.59	1.00
		Zm00001d014603	Peroxidase	0.27	1.35	0.95
