# Supplementary material for: Nitrate and Ammonium Affect the Overall Maize Response to Nitrogen Availability by Triggering Specific and Common Transcriptional Signatures in Roots
Source: Int J Mol Sci. 2020 Jan 20;21(2):686. doi: 10.3390/ijms21020686 (PMC7013554; doi:10.3390/ijms21020686)
Supplement: Supplementary file 1 [file ijms-21-00686-s001.zip › SUPPLEMENTARY MATERIALS_Ravazzolo_et_al_2019_IJMS/Table S7_S8.pdf]

Table S7. Complete quantification for all free amino acids detected as average of µg/g of weighted tissue ± SE and the proportion of total amino acids (%). **TOT**: calculated sum of total amino acids. **Δ% -N**: calculated percentage increase/decrease of total free amino acids detected in each treatment (+NO_3^- or +NH_4^+) with respect to the N-deficient treatment (-N). The asterisk indicates significant differences with respect to -N treatments (Student's t-test with: ** p≤0.01, * p≤0.05).

	-N ROOT			+NO_3^- ROOT				+NH_4^+ ROOT				-N LEAVES			+NO_3^- LEAVES				+NH_4^+ LEAVES			
**FREE AA**	**µg/g**	**SE**	**%**	**µg/g**	**SE**	**%**	**p**	**µg/g**	**SE**	**%**	**p**	**µg/g**	**SE**	**%**	**µg/g**	**SE**	**%**	**p**	**µg/g**	**SE**	**%**	**p**
GLY	1.72	0.30	1.53	1.73	0.28	1.77		2.62	0.27	2.45		1.53	0.29	1.82	1.99	0.21	1.85		2.46	0.21	1.30	
ALA	28.08	0.19	25.06	20.15	0.15	20.57	*	22.01	0.21	20.56	*	14.72	0.15	17.58	23.23	0.19	21.58	*	40.65	0.37	21.58	*
SER	2.04	0.29	1.82	1.19	0.27	1.22	**	1.28	0.35	1.20	**	3.53	0.27	4.22	6.38	0.42	5.93	**	6.33	0.42	3.36	**
PRO	1.70	0.17	1.52	2.45	0.13	2.50	**	1.19	0.09	1.11	**	3.03	0.18	3.62	5.03	0.16	4.67	**	9.35	0.12	4.97	**
VAL	12.88	0.52	11.50	11.25	0.47	11.49	**	14.20	0.58	13.26	**	8.93	0.41	10.66	14.06	0.69	13.06	**	23.99	0.92	12.74	**
THR	2.05	0.12	1.83	2.09	0.12	2.14	**	2.46	0.19	2.30	**	1.21	0.18	1.45	1.56	0.19	1.45	**	4.23	0.23	2.24	**
CYS	0.76	0.06	0.68	0.44	0.06	0.45		0.12	0.00	0.11		0.43	0.03	0.51	0.42	0.03	0.39		0.75	0.09	0.40	
LEU/ILE	16.13	0.73	14.40	15.61	0.54	15.94	**	8.49	0.29	7.93	**	2.87	0.27	3.43	3.64	0.37	3.38	**	9.15	0.60	4.86	**
ASN	7.31	0.44	6.52	0.93	0.17	0.95	*	6.90	0.44	6.44	*	9.39	0.58	11.21	12.57	1.04	11.68	*	29.68	2.19	15.76	*
ASP	1.99	0.19	1.78	1.89	0.27	1.93		1.56	0.31	1.46		1.97	0.42	2.35	3.02	0.67	2.81		7.04	0.71	3.74	
GLN	0.91	0.18	0.81	0.49	0.14	0.50	*	0.21	0.04	0.19	*	0.46	0.06	0.55	0.37	0.06	0.35	*	0.66	0.19	0.35	*
GLU	3.79	0.39	3.38	6.38	0.48	6.51	**	11.28	1.21	10.54	**	6.24	12.90	7.45	8.56	12.77	7.95	**	14.00	0.55	7.44	**
LYS	0.60	0.08	0.53	0.24	0.07	0.25		0.53	0.08	0.50		0.50	0.08	0.59	0.22	0.06	0.21		1.40	0.08	0.74	
MET	5.83	0.54	5.20	4.77	0.80	4.87	**	5.64	1.42	5.27	**	9.53	1.06	11.38	6.39	0.39	5.94	**	22.15	1.52	11.76	**
HIS	1.44	0.08	1.28	4.19	0.21	4.28	**	0.63	0.17	0.59	**	0.48	0.10	0.57	0.50	0.13	0.47	**	0.42	0.06	0.22	**
PHE	2.26	0.32	2.02	2.04	0.45	2.08		1.35	0.24	1.26		5.37	0.69	6.41	4.19	0.78	3.90		5.65	0.98	3.00	
ARG	2.83	0.15	2.53	4.60	0.23	4.69	**	3.60	0.22	3.37	**	0.12	0.07	0.14	0.13	0.05	0.12	**	0.15	0.06	0.08	**
TYR	18.81	1.25	16.78	16.66	0.79	17.01	**	21.91	1.10	20.46	**	12.81	0.72	15.29	14.63	0.75	13.59	**	9.79	0.62	5.20	**
TRP	0.94	0.05	0.84	0.83	0.04	0.85	**	1.10	0.05	1.02	**	0.64	0.04	0.76	0.73	0.03	0.68	**	0.49	0.03	0.26	**
**TOT**	**112.06**	1.70	100	**97.94**	1.37	100		**107.06**	1.56	100		**83.75**	1.02	100	**107.62**	1.42	100		**188.34**	2.55	100	
**Δ% -N**						-12.6%				-4.5%							+28.5%				+124.9%	

Table S8. Complete quantification for all hydrolysed amino acids detected as average of µg/g of weighted tissue ± SE and the proportion of total amino acids (%). **TOT**: calculated sum of total amino acids. **Δ% -N**: calculated percentage increase/decrease of total hydrolysed amino acids detected in each treatment (+NO_3^- or +NH_4^+) with respect to the N-deficient treatment (-N). The asterisk indicates significant differences with respect to -N treatments (Student's t-test with: ** p≤0.01, * p≤0.05).

	-N ROOT			+NO_3^- ROOT				+NH_4^+ ROOT				-N LEAVES			+NO_3^- LEAVES				+NH_4^+ LEAVES			
**HYDR.AA**	**µg/g**	**SE**	**%**	**µg/g**	**SE**	**%**	**p**	**µg/g**	**SE**	**%**	**p**	**µg/g**	**SE**	**%**	**µg/g**	**SE**	**%**	**p**	**µg/g**	**SE**	**%**	**p**
GLY	57.30	0.90	0.70	41.68	0.96	0.63	**	29.96	1.54	0.39	**	28.15	1.15	0.44	18.53	0.58	0.43	**	15.81	0.77	0.76	**
ALA	1042.81	2.50	12.68	390.80	1.73	5.92	**	569.89	1.35	7.45	**	228.59	1.15	3.54	112.12	1.54	2.57	**	80.18	1.54	3.85	**
SER	100.63	3.66	1.22	69.68	2.69	1.06	**	52.01	1.69	0.68	**	16.25	1.35	0.25	13.07	0.96	0.30	**	9.25	0.96	0.44	**
PRO	607.75	15.20	7.39	573.04	14.4	8.68	**	395.29	14.24	5.17	**	191.54	8.47	2.96	183.43	7.51	4.21	**	70.01	8.47	3.36	**
VAL	296.47	12.32	3.61	218.82	9.24	3.32	**	100.84	7.31	1.32	**	125.04	5.97	1.93	96.78	5.39	2.22	**	22.17	5.20	1.06	**
THR	122.03	6.74	1.48	123.47	6.16	1.87	**	53.92	2.89	0.70	**	39.82	1.44	0.62	34.67	1.35	0.80	**	12.03	1.54	0.58	**
CYS	3280.00	14.63	39.88	3024.74	13.4	45.83	*	4927.87	14.05	64.40	*	4474.61	7.51	69.22	2559.45	4.23	58.73	*	1327.59	5.39	63.71	*
LEU/ILE	673.41	11.74	8.19	530.62	12.3	8.04	**	303.73	7.89	3.97	**	288.61	4.62	4.46	291.66	3.27	6.69	**	106.46	2.12	5.11	**
ASP	36.47	1.15	0.44	29.97	1.15	0.45	**	9.93	0.77	0.13	**	6.89	0.58	0.11	10.43	0.64	0.24	**	1.59	0.13	0.08	**
ASP.a	23.04	0.58	0.28	16.13	0.31	0.24	**	4.85	0.12	0.06	**	3.91	0.27	0.06	5.56	0.25	0.13	**	0.93	0.06	0.04	**
GLU	44.65	0.60	0.54	20.05	0.77	0.30	*	9.51	0.44	0.12	*	17.13	0.50	0.27	18.89	0.38	0.43	*	13.08	0.31	0.63	*
LYS	8.05	0.17	0.10	4.28	0.17	0.06	**	2.18	0.12	0.03	**	3.97	0.13	0.06	3.87	0.06	0.09	**	2.67	0.10	0.13	**
MET	68.42	1.54	0.83	93.65	5.58	1.42	**	44.02	1.35	0.58	**	14.87	0.77	0.23	24.43	0.96	0.56	**	7.67	0.58	0.37	**
HIS	922.69	5.00	11.22	821.18	4.81	12.44	**	499.59	1.15	6.53	**	444.92	1.15	6.88	445.25	0.77	10.22	**	132.01	0.58	6.33	**
PHE	278.06	0.77	3.38	173.53	0.77	2.63	*	212.14	0.96	2.77	*	216.69	1.35	3.35	243.98	0.96	5.60	*	194.58	0.46	9.34	*
ARG	632.21	1.20	7.69	446.18	0.85	6.76	**	421.51	0.58	5.51	**	350.91	0.63	5.43	285.49	0.29	6.55	**	80.46	0.21	3.86	**
TRP	29.73	0.08	0.36	21.63	0.06	0.33	**	14.82	0.03	0.19	**	12.45	0.04	0.19	10.06	0.02	0.23	**	7.47	0.01	0.36	**
**TOT**	8223.72	187.3	100	6599.45	170	100		7652.066	275.3	100		6464.362	250.4	100	4357.65	143.1	100		2083.95	74.2	100	
**Δ% -N**						-19.7%				-6.9%							-32.6%				-67.8%	
