# Supplementary material for: Nitrate and Ammonium Affect the Overall Maize Response to Nitrogen Availability by Triggering Specific and Common Transcriptional Signatures in Roots
Source: Int J Mol Sci. 2020 Jan 20;21(2):686. doi: 10.3390/ijms21020686 (PMC7013554; doi:10.3390/ijms21020686)
Supplement: Supplementary file 1 [file ijms-21-00686-s001.zip › SUPPLEMENTARY MATERIALS_Ravazzolo_et_al_2019_IJMS/Table S9.pdf]

Table S9. Amino acid transitions for HILIC-MS/MS quantification.

Compound	Polarity	Q1	Q3	Capillary	Collision	Dwell Time
**Glycine**	Pos.	76.00	30.30	40.000	8.000	0.1
**Alanine**	Pos.	90.00	44.50	40.000	8.000	0.1
**Serine**	Pos.	106.00	60.40	30.000	8.500	0.1
**Proline**	Pos.	116.00	70.30	40.000	10.500	0.1
**Valine**	Pos.	118.00	72.40	30.000	8.500	0.1
**Threonine**	Pos.	120.00	74.40	30.000	8.000	0.1
**Cystine**	Pos.	122.00	75.70	30.000	11.500	0.1
**Isoleucine**	Pos.	132.00	86.50	40.000	7.000	0.1
**Leucine**	Pos.	132.00	86.50	40.000	7.500	0.1
**Asparagine**	Pos.	133.00	87.40	40.000	6.500	0.1
**Aspartic Acid**	Pos.	134.00	74.30	33.876	12.000	0.1
**Glutamine**	Pos.	147.00	84.40	30.000	14.000	0.1
**Lysine**	Pos.	147.10	84.40	30.000	13.500	0.1
**Glutamic Acid**	Pos.	148.00	83.70	30.000	13.000	0.1
**Methionine**	Pos.	150.00	133.40	38.318	6.000	0.1
**Histidine**	Pos.	156.10	110.00	40.000	9.500	0.1
**Phenylalanine**	Pos.	166.10	119.60	40.000	9.500	0.1
**Arginine**	Pos.	175.10	70.40	40.000	18.000	0.1
**Tyrosine**	Pos.	182.10	135.60	40.000	10.000	0.1
**Tryptophan**	Pos.	205.10	187.60	40.000	7.000	0.1
